# Separator Design for High‐Performance Aqueous Zinc‐Ion Batteries: Recent Advances and Future Outlooks

**DOI:** 10.1002/smsc.202500466

**Published:** 2025-11-27

**Authors:** Wenyi Guo, Jiashu Chen, Xinzhong Wang, Yiwen Su, Jingyu Sun, Guangping Zheng

**Affiliations:** ^1^ Research Institute for Advanced Manufacturing, and Department of Mechanical Engineering The Hong Kong Polytechnic University Hung Hom, Kowloon, Hong Kong P. R. China; ^2^ College of Energy Soochow Institute for Energy and Materials Innovations Jiangsu Provincial Key Laboratory for Advanced Carbon Materials and Wearable Energy Technologies Soochow University Suzhou 215006 P. R. China

**Keywords:** aqueous zinc ion batteries, separator engineering, Zn metal

## Abstract

Aqueous zinc‐ion batteries (AZIBs) have garnered increasing attention as promising candidates for large‐scale energy storage applications, owing to their safety, cost‐effectiveness, and high theoretical capacity. However, challenges such as dendrite formation, side reactions, and cathode dissolution continue to hinder their widespread adoption. As a critical component in direct contact with both the electrodes and electrolyte, the separator plays a significant role in determining the cycle life of the cell. This review provides a comprehensive review of recent strategies for separator modification, focusing on key approaches such as surface functionalization, regulation of porous structure, and the design of composite matrices. The review highlights the mechanisms by which these modifications influence ion transport, interface stability, and dendrite suppression. Additionally, it explores separator engineering technologies with promising practical applications, bridging the gap between fundamental research and real‐world implementation. It is suggested that separator engineering is not only a crucial pathway for enhancing battery performance but also an essential factor for transitioning AZIBs from laboratory‐scale research to industrial‐scale applications. By analyzing the structure‐property relationships of separator materials, this work aims to guide the rational design of next‐generation high‐performance separators and contribute to the practical deployment of zinc‐based energy storage technologies.

## Graphical abstract

Separators for high‐performance AZIBs warrant design considerations from multiple working mechanisms, thereby achieving electrode stabilization and expanding device applications. Furthermore, to advance the commercialization of AZIBs, the practical application potential of the separator also needs to be evaluated and considered. Following this guideline, it is hopeful that a promising separator for aqueous cell can be manufactured.

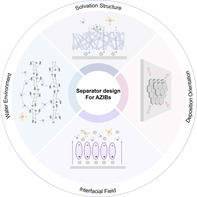

## 1 Introduction

In light of the continuous growth in global energy consumption and the increasingly severe environmental impacts, particularly rising carbon emissions, the development of environmentally friendly renewable energy systems to mitigate the depletion of fossil energy sources has to be prioritized.^[^ ^1^, ^2^ ^]^ In contrast, renewable energy represented by solar, wind, and other energy sources offer clean advantages.^[^ ^3^ ^]^ Nevertheless, their inherent safety issues and economic limitations hinder potential applications in large‐scale energy storage systems.^[^ ^4^, ^5^ ^]^ Among existing energy storage technologies, lithium‐ion batteries (LIBs) currently dominate applications in portable electronics and electric vehicles. Nevertheless, their widespread adoption in large‐scale energy storage systems is hindered by several inherent limitations: 1) The flammability of organic electrolytes presents significant risks; 2) the limited abundance of lithium resources in the earth's crust leads to high costs; and 3) the environmental toxicity of electrodes and electrolyte materials complicates recycling processes.^[^ ^6^ ^]^ Comparatively, aqueous zinc ion batteries (AZIBs) have demonstrated promising potential for energy storage by virtue of the high theoretical capacity of Zn metal (820 mAh g^−1^), suitable redox potential (–0.76 V vs. standard hydrogen electrode), and abundant crustal reserves.^[^ ^7^, ^8^ ^]^ Meanwhile, the application of water electrolyte endows AZIBs with the safe and environmental‐friendly characteristics.

**Figure 1:**
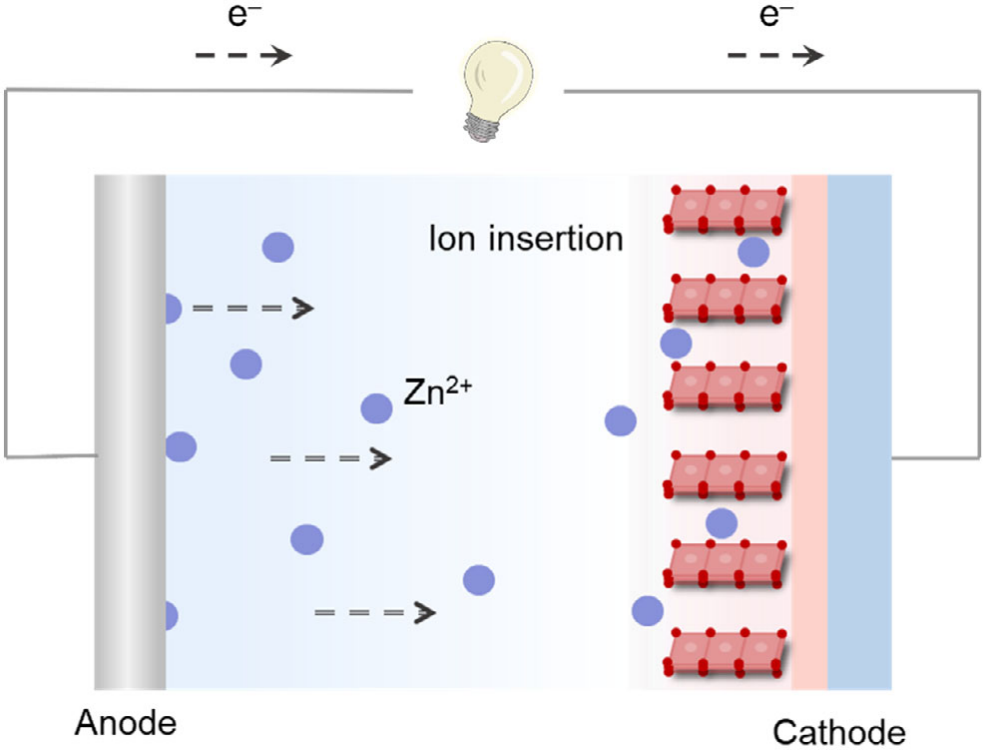
Demonstration of the basic working mechanism of AZIBs.

As illustrated in **Figure** **1**, the operational mechanism of AZIBs relies on the reversible electrochemical shuttling of Zn^2+^ between the cathode and anode.^[^ ^9^ ^]^ During charging processes, Zn^2+^ undergo electrochemical deposition onto the anode surface, while discharge processes involve the reversible platting of Zn, accompanied by electron transfer through the external circuit. In the case of the cathode, Zn^2+^ tends to undergo repeated storage processes through various mechanisms, including intercalation and transformation. It is worth noticing that despite their intrinsic advantages, the current AZIBs system is still confronted with several critical challenges.^[^ ^10^ ^]^ One primary issue is the inherent microscopic protrusions presented on the surface of commercial Zn foil anodes, which promote localized electric field intensification.^[^ ^11^ ^]^ This phenomenon results in non‐uniform Zn deposition, ultimately leading to dendrite formation. Such dendrites not only risk separator penetration and internal short circuits but also accelerate hydrogen evolution process due to their high surface area.^[^ ^12^ ^]^ Additionally, performance deficiencies in the separator, such as uneven pore distribution and insufficient interfacial stability, further contribute to ion flux imbalance and the occurrence of side reactions. Moreover, the cathode material is susceptible to encounter dissolution or structural collapse during cycling, primarily due to the combined influence of highly active water molecules and the repeated intercalation and de‐intercalation of Zn^2+^.^[^ ^13^, ^14^ ^]^ This degradation manifests as active material dissolution or lattice collapse, causing rapid capacity fading and reduced cycle life of the full cell.^[^ ^15^ ^]^

To address these challenges, ongoing efforts have focused on structural optimization of cathode materials and stabilization of anode interfaces, aiming to develop dendrite‐free and side‐reaction‐suppressed AZIBs through strategic modulation of cationic behavior, water molecule activity, and interfacial properties.^[^ ^16^, ^17^, ^18^ ^]^ It has been proved that enhancing the mechanical strength of active materials or mitigating the erosive effects of water molecules through interfacial engineering and structural design can substantially enhance the performance of cathode during long‐term cycling. In parallel, the refinement of the electrolyte components or the deposition behavior is of vital significance for improving the cycle life of Zn anode.^[^ ^19^ ^]^ For instance, Wang et al. suppressed interfacial side reactions by incorporating 20 M LiTFSI into the electrolyte, employing a high salt concentration to decouple the pristine hydrated structure of the cation, thereby constructing a “water in salt” environment.^[^ ^20^ ^]^ In addition, modification of the Zn anode via the covalent organic framework interfacial layer is capable of reducing the surface energy of the Zn(002) plane and realizes the orientation growth during plating process.^[^ ^21^ ^]^ Notably, while significant progress has been made in optimizing the electrodes and electrolyte, the separator remains substantial potential for further improvement. During the series of electrochemical processes mentioned above, the separator plays a critical role in enabling ionic conduction through its porous structure. More importantly, it prevents direct electron transfer between the electrodes, thereby ensuring the stable operation of the cell. Consequently, the development of advanced separators represents a promising avenue for improving overall AZIB performance, with particular significance for enhancing safety protocols and extending cycle life.

Despite numerous advancements in the optimization of separators, fundamental research challenges related to zinc‐based energy storage still require a comprehensive classification and generalization of existing studies. Furthermore, the industrial application assessment systems that limit the broader use of zinc‐based energy storage devices remain underdeveloped. This review first identifies the key research bottlenecks in Zn‐based energy storage systems. Therefore, this review analyzes the research bottlenecks of AZIBs that hinder their future application. On this basis, we comprehensively evaluate research strategies employing various separator substrates. Subsequently, according to the distinct operational mechanisms of the state‐of‐the‐art reports, we summarize and conclude the application of separator engineering in the stabilization of AZIBs. Ultimately, we provide insights into future development directions, focusing on material design, characterization methods, and fabrication processes that offer theoretical guidance and technical references for the advancement of high‐performance AZIBs separators.

## 2 Critical Bottlenecks

Although zinc‐based energy storage devices offer numerous advantages, their practical applications still face critical challenges. The inhomogeneous deposition behavior of metallic Zn, coupled with side reactions induced by highly reactive solvent molecules, constrains the performance enhancement and practical application of AZIBs.

### 2.1 Hydrogen Evolution Reaction

Thermodynamically, the standard electrode potential of Zn is significantly lower than that of water, contributing to the spontaneous reaction of Zn with water in a weakly acidic or neutral electrolyte such as ZnSO_4_ and Zn(OTf)_2_. This reaction not only generates hydrogen gas and poses a safety hazard, but also causes continuous corrosion of Zn, severely degrading the cycling stability of the battery.

**Figure 2:**
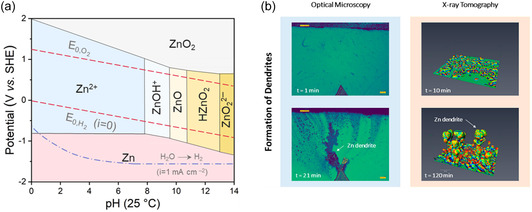
a) Pourbaix diagram. Reproduced with permission.^[^ ^23^ ^]^ Copyright 2024, Elsevier. b) In situ optical microscopy imaging and X‐ray tomography of Zn deposition. Reproduced with permission.^[^ ^30^ ^]^ Copyright 2023, American Chemical Society.

The consumption of H^+^ by the hydrogen evolution reaction (HER) leads to an increase in OH^‐^ concentration at the electrode/electrolyte interface, which in turn reacts with Zn^2+^ and other electrolyte components to form insulating byproducts.^[^ ^22^ ^]^ The accumulation of these byproducts obstructs uniform deposition and increases interfacial impedance. It is worth noting that theoretically, the deposition potential of Zn is much lower than the HER potential in the whole pH range. However, for actual electrochemical processes, the presence of an overpotential causes the onset potential of the HER to shift negatively, resulting in a delayed reaction relative to Zn deposition (**Figure** **2**a).^[^ ^23^ ^]^

Due to the weak ionization of water molecules, the resulting H_3_O^+^ diffuse and migrate toward the electrode surface, subsequently participating in HER.^[^ ^24^ ^]^ This reaction comprises the following sequential steps:^[^ ^25^ ^]^

First, H_3_O^+^ are reduced by electron transfer at the electrode surface to form adsorbed hydrogen atoms

(1) $$H_{3}O^{+}+{\text{ e}}^{-}\to \text{ H}+H_{2}O*\;\left(\text{Volmer reaction}\right)$$

Subsequently, H_3_O^+^ in the solution react with adsorbed atom or combine with adjacent adsorbed hydrogen atoms

(2) $$H_{3}O^{+}+{\text{ e}}^{-}\;+\text{ H}\to H_{2}+{\text{ H}}_{2}O*\;\left(\text{Heyrovsky reaction}\right)$$

(3) $$H+H\to H_{2}**\;\left(\text{Tafel reaction}\right)$$

Ultimately, the generated H_2_ molecules desorb from the electrode surface and diffuse into the electrolyte, where they form bubbles and escape.

Within this sequence of events, the rate‐determining step is generally considered to be either the recombination of hydrogen ions with electrons to discharge, or the process whereby hydrogen atoms combine to form molecules.

### 2.2 Dendrite Growth

The growth of dendrite is one of the most critical challenges for Zn anode, which involves complex electrochemical processes and multiscale interfacial evolution. From a microstructural point of view, the poor mechanical contact between vertically growing dendrites and the substrate can easily lead to the detachment of active materials, evolving into electrochemically deactivated “dead Zn”.^[^ ^26^, ^27^ ^]^ This leads to a reduction in the effective use of the active material, thereby weakening the performance of the cell. Furthermore, the increased specific surface area of the dendrites provides more sites for side reactions, which accelerates electrolyte consumption and corrosion processes. These combined factors contribute to irreversible loss of active material and electrolyte, significantly lowering the coulombic efficiency and reversible capacity of AZIBs.

During deposition, the reduction of Zn^2+^ involves successive kinetic steps: ion diffusion, electrochemical reduction, nucleation, and crystal growth. Kinetic studies indicate that deposition rate is mainly influenced by the electrochemical polarization, while the electric double layer effect on the Zn surface significantly amplifies the local electric field strength. Specifically, the intrinsic roughness of commercial Zn foils results in the formation of micro‐scale protrusions with small radii of curvature.^[^ ^28^ ^]^ In addition, adsorbed ions tend to undergo 2D diffusion, preferentially reducing at energetically favorable sites to form protrusions.^[^ ^29^ ^]^ These protrusions further attract Zn^2+^ aggregation through the well‐known “tip effect,” inducing subsequent carriers to preferentially reduce at the existing protrusions for minimizing surface energy and exposure area. Over successive cycles, these protrusions evolve into dendritic structures, which are a primary mechanism for anode failure (Figure 2b).^[^ ^30^ ^]^

### 2.3 Corrosion and Passivation

The corrosion of AZIBs primarily arises from the combined effects of chemical self‐discharge corrosion and electrochemical corrosion.^[^ ^31^, ^32^ ^]^ Among them, self‐corrosion is a spontaneous process driven by chemical potential, while electrochemical corrosion results from differences in electrode potential, which are integral to the overall reaction mechanism of the cell. Compared with alkaline electrolytes, neutral or weakly acidic electrolytes are significantly more effective in mitigating self‐corrosion. However, corrosion phenomena persist on the Zn metal surface, mainly due to the intrinsic grain boundary corrosion inherent to Zn. This type of corrosion generates microscopic galvanic cells at the grain boundaries, which initiate localized electrochemical corrosion reactions.^[^ ^33^ ^]^ The corrosion process typically begins with the formation of surface pits, which progressively propagate along the grain boundaries, eventually leading to the failure of the electrode structure.

Moreover, during the charge‐discharge cycles of AZIBs, the Zn anode frequently undergoes surface passivation, in parallel with corrosion and hydrogen evolution reactions. This passivation is triggered by localized increases in OH^−^ concentration, causing Zn^2+^ to react with OH^−^ and form insulating byproduct layers such as ZnO/Zn(OH)_2_ on the electrode surface.^[^ ^34^, ^35^ ^]^ The formation of this passivation layer not only significantly reduces the anode's conductivity but also increases the interfacial transfer impedance and reduces the availability of active nucleation sites for Zn deposition.

## 3 Separator Engineering

The performance of AZIBs is severely constrained by the inherent structural defects of conventional glass fiber (GF) separators. Specifically, its non‐uniform pore distribution leads to an imbalance in Zn^2+^ flux, triggering excessive local current density. This, in turn, promotes uneven Zn deposition and dendrite growth. Furthermore, the unavoidable HER on the surface of the electrode during electrochemical cycling introduces several detrimental effects, complicating the overall performance and stability of the battery.

### 3.1 Design Objectives of Ideal Separator

As a multifunctional component, the separator serves to prevent direct contact between the cathode and anode, facilitates the transport of the electrolyte, and aids in ion migration.^[^ ^36^ ^]^ The physicochemical properties of the separator are therefore crucial in determining the cycle life and overall performance of the battery. Meanwhile, a well‐designed separator can also enhance the full performance potential of both the cathode and anode materials. In general, an ideal separator should exhibit long‐term chemical and thermodynamic stability in aqueous electrode solutions, preventing structural collapse due to the erosion of water molecules or electrode reactions.^[^ ^37^ ^]^ Furthermore, the separator should also possess excellent mechanical strength and flexibility to resist dendrite growth and accommodate electrode deformation and volume expansion during cycling. Appropriate hydrophilicity, on the other hand, is essential to enable the separator to function as an effective electrolyte carrier, promoting ionic conduction. Additionally, key indicators such as porosity and charging properties of the separator are also worthy of attention. Altogether, through the modulation of electric and ionic fields, as well as the manipulation of free water molecules and solvation structures, an ideal separator can effectively regulate disordered metal deposition and minimize unfavorable side reactions.

According to existing research, adding hydrogen bond disruptors to electrolytes can weaken intermolecular forces between water molecules.^[^ ^18^, ^38^ ^]^ Meanwhile, the hydrophilic/hydrophobic design or functional group modification of the separator can further capture free water molecules through intermolecular interactions. This synergistic electrolyte‐separator strategy significantly reduces interfacial water activity, resulting in a pronounced negative shift in hydrogen evolution overpotential and effectively retarding the kinetics of the hydrogen evolution reaction. Concurrently, the confinement effect generated by nanopores on the separator surface synergizes with the coordination ability of anions in the electrolyte, substantially lowering the desolvation energy barrier fo Zn^2+^ and reducing bound water accumulation at the electrode interface. Specific surface‐modified functional groups achieve ion‐selective transport through electrostatic interactions, promoting directed Zn^2+^ migration while effectively repelling corrosive anions, thereby suppressing side reactions. Furthermore, separators with polar functional groups or composite separators modified with high‐dielectric‐constant materials can homogenize the interfacial electric field distribution, reduce Zn nucleation overpotential, and induce preferential growth of specific crystal planes.

### 3.2 Modification of Glass Fiber Separators

**Figure 3:**
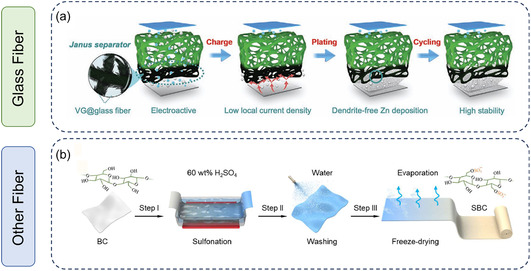
a) Illustration of the generation of the Janus separator combined with one‐side directly grown VG carpet. Reproduced with permission.^[^ ^39^ ^]^ Copyright 2020, Wiley‐VCH. b) Scalable production of the cellulose separator. Reproduced with permission.^[^ ^42^ ^]^ Copyright 2025, Elsevier.

Conventional GF separators often exhibit poor mechanical properties, non‐uniform pore distribution, and excessive thickness. Therefore, modifications involving the incorporation of functional materials (e.g., MXene, COF, ZIF, graphene, etc.) or the introduction of functional groups, such as amines and sulfonates, are necessary to enhance the electrochemical performance of the battery. For example, Li et al. employed chemical vapor deposition to in situ grow vertical graphene (VG) on GF, achieving single‐sided VG loading via plasma treatment (**Figure** **3**a).^[^ ^39^ ^]^ The 3D framework structure constructed by VG induced a homogenized electric field distribution, while the oxygen and nitrogen heteroatoms introduced during the plasma treatment further enhanced the zincophilicity of the modified separator. In addition, Nan et al. modified GF separators with polytriazolylbenzimidazole (PTIB) enriched with imidazole groups. The strong coordination of imidazole groups with Zn^2+^ not only facilitated ionic transport, but also significantly reduced the Zn^2+^ concentration gradient at the interface. In terms of the inhibition of side‐reaction activity, the porosity of porous materials such as metal‐organic frameworks has been reported to provide an effective guideline for constraining the activity of water molecules at the interface.^[^ ^40^ ^]^ When compounded with GF separators, these materials are expected to construct specialized channels with near single‐ion conduction, reducing the accumulation of water at the metal/electrolyte interface. These modification strategies offer promising solutions to key technological challenges in AZIBs by precisely tailoring the physicochemical properties of the separators.

### 3.3 Creation of Self‐Supporting Separators

In addition to commercial GF separators, cellulose‐based separators have demonstrated advantages for AZIBs due to their intrinsic structural properties.^[^ ^41^ ^]^ As a natural polymer material extracted from living organisms, cellulose is environmentally friendly, cost‐effective, and recyclable, rendering it well‐suited for aqueous Zn‐based energy storage devices (Figure 3b).^[^ ^42^ ^]^ Notably, owing to the strong hydrogen bonding interactions between cellulose, cellulose‐based separators are capable of providing excellent anti‐swelling properties and adjustable mechanical strength for batteries. Moreover, the abundance of polar functional groups in cellulose enables interactions with solvent water molecules and facilitates the modulation of cation migration.^[^ ^43^, ^44^, ^45^ ^]^ With further design and modification, these groups also facilitate chemical modification. By introducing functional groups such as carboxyl and sulfonic acid, the ionic conductivity and Zn^2+^ distribution of cellulose separators can be significantly improved, addressing issues such as dendrite growth and anode corrosion. Meanwhile, in terms of synthesizing polymer separators, polyacrylonitrile‐based (PAN‐based) materials have attracted much attention due to their regular molecular structure and abundant cyano‐functional groups.^[^ ^46^ ^]^ These separators exhibit remarkable mechanical properties, while their uniformly distributed nanoscale pores work synergistically with their inherent zincophilic to promote uniform deposition and effectively suppress dendrite formation. In contrast, polypropylene (PP) separators, although known for their excellent chemical and thermal stability, often face challenges in metal deposition due to their inherently hydrophobic surface and micron‐scale pore distribution.^[^ ^47^ ^]^ As a result, surface modification to enhance zincophilic is typically required for practical applications. Additionally, Nafion separators, characterized by their exceptional mechanical strength and dense pore structure, provide an effective physical barrier against dendrite penetration.^[^ ^48^ ^]^ As a cation‐exchange membrane, the abundant sulfonic acid groups (—SO_3_H) within Nafion not only facilitate selective Zn^2+^ transport and improve ion mobility, but also suppress side reactions by modulating local pH at the interface, thereby enhancing the cycling stability of the cell.^[^ ^49^, ^50^ ^]^ Each polymer‐based separator offers distinct advantages, presenting a variety of materials that are well‐suited for diverse applications in AZIBs. Each of these polymer‐based separators has its own unique characteristics, providing a diverse selection of materials for the development of zinc‐based batteries for different application scenarios.

## 4 Optimization Mechanisms Based on Separator Design

### 4.1 Field Management

The separator, as the carrier of the electrolyte, plays a crucial role in the interaction with solvent molecules and serves as a key factor in regulating the components of the electrolyte. Ions in the electrolyte tend to be transported and diffused alongside the pores of the separator. However, the pore diameter of the traditional GF separator often exhibits large and non‐uniform pore sizes, which tends to induce a disordered metal deposition pattern. Therefore, researchers have started from the design of the separator to extend the service life of the Zn anode by rationally regulating the transport diffusion of Zn^2+^ in the diaphragm and the distribution of the electric field inside the diaphragm through considering various environmental factors, including the thermal, electric, and ionic fields.^[^ ^51^, ^52^, ^53^ ^]^

**Figure 4:**
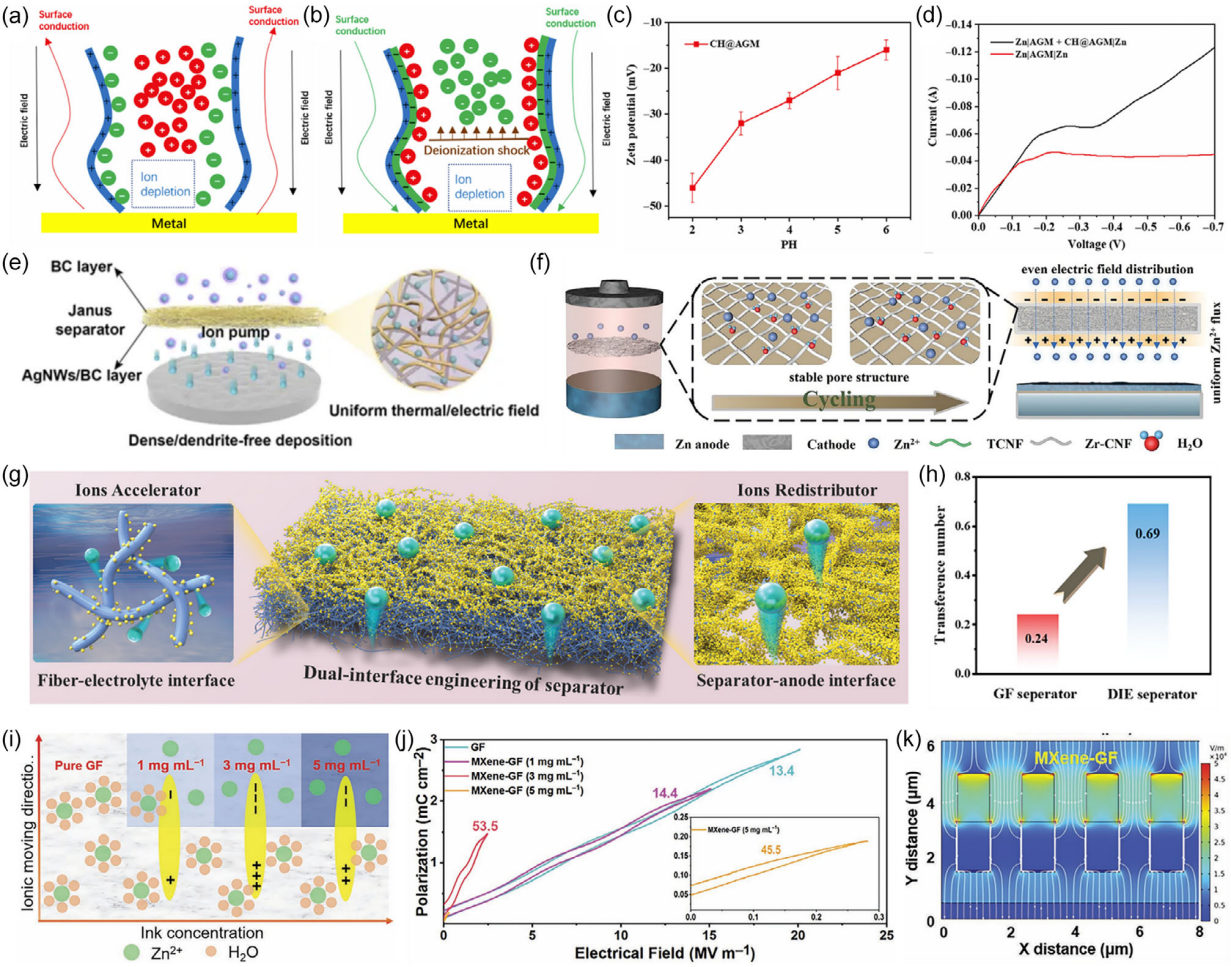
a,b) The influence of charged pores for surface conduction on metal electrodeposition. c) Zeta potential of CH@AGM separator. d) Voltammetry of symmetric cells in the ZnSO_4_ electrolyte. Reproduced with permission.^[^ ^54^ ^]^ Copyright 2020, American Association for the Advancement of Science. e) Scheme illustrating the function mechanism of AgNMWs/BC separator. Reproduced with permission.^[^ ^57^ ^]^ Copyright 2023, Wiley‐VCH. f) Zn^2+^ migration and Zn deposition behavior within the Zr‐CNF separator. Reproduced with permission.^[^ ^61^ ^]^ Copyright 2023, Wiley‐VCH. g) The schematic diagram of BTO decorated separator. h) Transfer number of GF and Janus separator. Reproduced with permission.^[^ ^62^ ^]^ Copyright 2022, Wiley‐VCH. i) The polarized charge distribution. j) Polarization‐electric field loops of different separators. k) Simulation of electrical field distribution for MXene‐GF. Reproduced with permission. Copyright 2022, Wiley‐VCH.

Given the porous nature of the separator, metal reduction above a critical voltage tends to prevent aggregation within the separator's pore structure. Instead, due to electroosmotic flow, the metal ions accumulate at the pore center, leading to dendrite growth (**Figure** **4**a). On this basis, Zhi et al. modified the absorbed glass mat (AGM) membrane by coating it with collagen hydrolysate (CH), which stabilizes the metal electrode by regulating the charge properties of the separator.^[^ ^54^ ^]^ As shown in Figure 4b, the oppositely charged Zn^2+^ ions in the pores provide surface conductivity to sustain electrodeposition, facilitating bulk diffusion when current flows through the depletion region. Meanwhile, SO_4_^2−^ is repelled by the electric field, promoting a more homogeneous distribution of ionic flux. Zeta potential tests confirm that CH molecules are negatively charged in aqueous solutions, enabling electrostatic self‐assembly with the positively charged AGM fibers, resulting in stable adsorption (Figure 4c). Current‐voltage measurements reveal that the surface charge in the porous medium significantly enhances or suppresses the over‐limiting current between the metal electrodes, thereby controlling metal electrodeposition behavior at high currents density (Figure 4d). Meanwhile, the combination of CH with Zn^2+^ extends the presence of cations and further extends the distribution of cations over the entire surface of the metal anode, guiding the growth of Zn metal along the horizontal direction of the anode. Similarly, molecules such as MXene, functional supramolecules, or graphene have been incorporated into commercial GF separators to modulate ion distribution.^[^ ^55^, ^56^ ^]^ As shown in Figure 4e, Zheng et al. prepared a Janus separator using green and nontoxic bacterial cellulose (BC) as the substrate and loaded it with silver nanowires (AgNWs).^[^ ^57^ ^]^ The uniformly distributed silver nanowires on one side of the separator enhance zincophilicity, serving as ion pumps to improve ion transport kinetics. Compared with the unmodified cellulose separator, the specific Janus separator corresponded to a significant increase in ionic conductivity, indicating its effective enhancement of cation diffusion kinetics. Additionally, the uniform electric and thermal fields generated by the AgNWs promote uniform Zn nucleation and deposition. As a result, the symmetric cell based on the modified AgNWs separator presented ideal thermal stability, with 920 stable cycles even at 80 °C.

High dielectric constant materials such as Nb_2_O_5_, ZnTiO_3_, and ZrO_2_, which induce Maxwell‐Wagner polarization, exhibit the potential guide ion migration in an orderly manner and' optimize metal deposition by regulating the interfacial electric field.^[^ ^58^, ^59^, ^60^ ^]^ In general, typically suffered from solvation and internal fiber structure degradation due to hydrogen bond rearrangement over extended use, the metal deposition cannot be well controlled. However, by hydrolyzing cellulose with Zr^4+^, a Zr—O crosslinked coating is formed on the surface of the separator, preventing swelling and deformation during long‐term operation (Figure 4f).^[^ ^61^ ^]^ The amorphous Zr—O coating creates a directional electric field at the interface through Maxwell–Wagner polarization, reducing nucleation overpotential and enhancing ion transport kinetics. In this way, the homogenized Zn^2+^ flux contributes to uniform metal deposition, thus prolongs the operation lifetime of the Zn||Zn symmetric cell. Similarly, Liang et al. modified BTO on glass fiber separators by vacuum filtration, while enriching them near the anode surface (Figure 4 g).^[^ ^62^ ^]^ This graded concentration distribution helps regulate the fiber/electrolyte interface and the separator/anode interface, respectively. The spontaneous polarization effect and zincophilicity of BTO facilitate ion transport, while the decorated separators help redistribute ion flux and homogenize the electric field at the anode interface. Combined with impedance and contact angle tests, it can be seen that the migration number of the symmetric cell can be improved from 0.24 to 0.69 for the GF counterpart after the introduction of BTO molecules (Figure 4h). In addition, Su et al. sprayed conductive MXene nanosheets on one side of a commercial GF separator.^[^ ^63^ ^]^ As depicted in Figure 4i, the cross‐sectional view of the polarized charge distribution indicates that the MXene layer generates a built‐in directional electric field. According to dielectric constant measurements, the MXene‐modified separators exhibited higher values (53.5) of dielectric constant (ε) than the unmodified GF (Figure 4j). This enhancement facilitates ion migration, reducing the dynamic differences between redox reactions and mass transfer, thereby mitigating dendrite formation. The optimization of the electric field in the vicinity of Zn by MXene‐GF is further confirmed by the distribution of the electric field simulated by the finite element model, as shown in Figure 4k. The modified separator corresponds to an average intensity of 9841.9 V m^−1^, which is significantly higher than that of its counterpart (6519.9 V m^−1^), enhancing ion migration to retard the formation of dendrites.

### 4.2 Solvation Structure Regulation

In the operation of AZIBs, water plays a significant role as the solvent of the electrolyte, directly affecting the stability of metallic Zn. In general, due to the strong correlation between Zn^2+^ and water, Zn^2+^ tends to coordinate with six water molecules to undergo coordination to form a hydrated structure ([Zn(H_2_O)_6_]^2+^), which undermines the stability of the bound water through the M—OH bond.^[^ ^64^, ^65^ ^]^ When the solvated cations migrate to the electrode surface, the desolvation process occurs in the electric double layer and removes the bound water.^[^ ^66^, ^67^ ^]^ During this process, harmful water molecules tend to accumulate on the metal/electrolyte interface and eventually cause serious hydrogen precipitation and corrosion problems, accelerating dendrite growth while decreasing the utilization rate of active Zn.^[^ ^16^ ^]^

**Figure 5:**
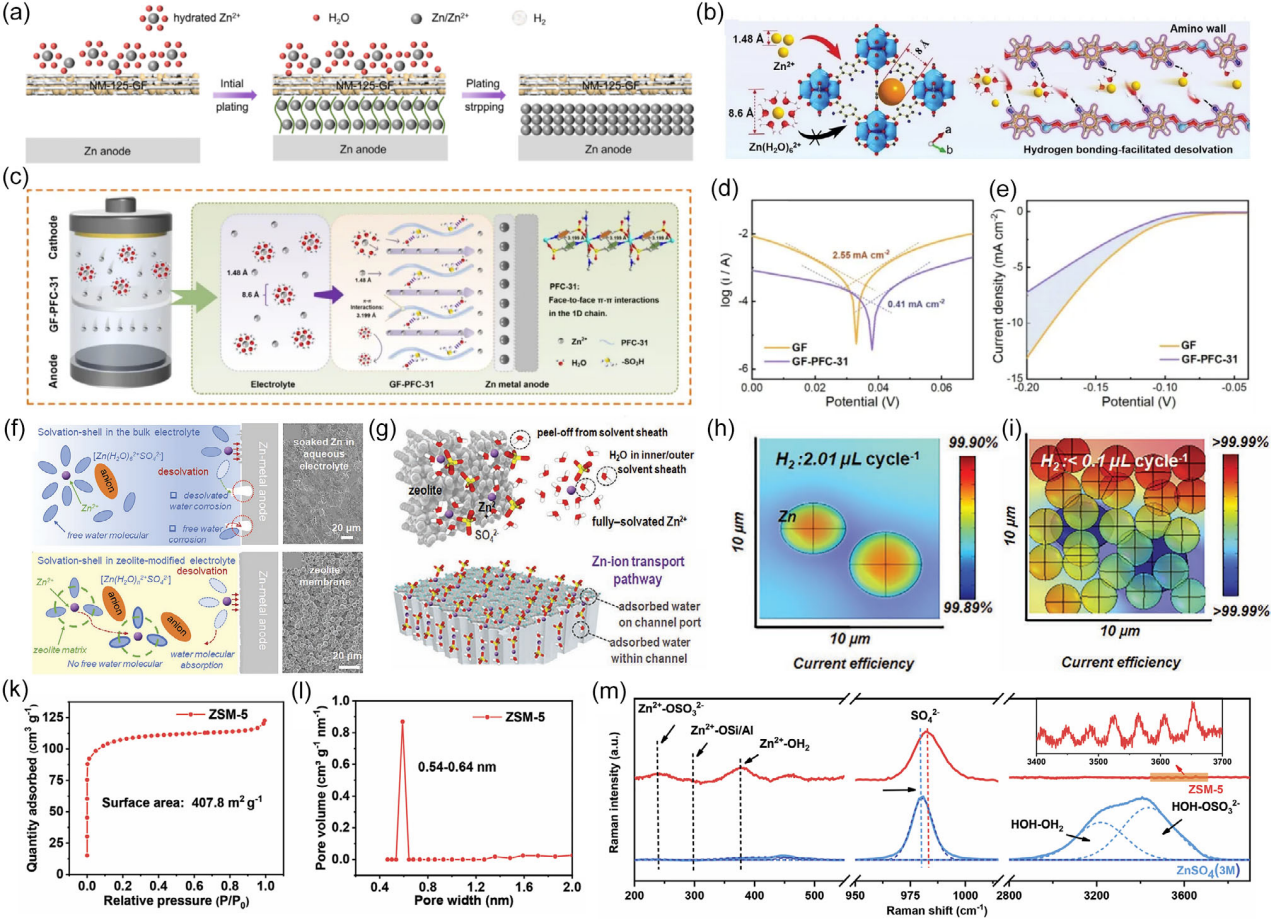
a) Zn deposition mechanism for NM‐125 separator. Reproduced with permission.^[^ ^71^ ^]^ Copyright 2025, Elsevier. b) The fundamental mechanism of UiO‐66‐NH_2_ in modulating anion ions. Reproduced with permission.^[^ ^72^ ^]^ Copyright 2023, Wiley‐VCH. c). Mechanism illustrating the deposition processes for Zn anodes with the application of GF‐PFC‐31. d) Tafel and e) Linear scanning voltammetry test for cell with GF and GF‐PFC‐31 separator. Reproduced with permission.^[^ ^73^ ^]^ Copyright 2024, Elsevier. f) Schematic illustration of water molecule decomposition process on metal/electrolyte. g) Zn^2+^ transport mechanism within the zeolite molecular sieve membrane. h,i) Current efficiency simulation for determining H_2_ evolution. Reproduced with permission.^[^ ^76^ ^]^ Copyright 2021, Wiley‐VCH. k) Nitrogen adsorption/desorption isotherm and l) the inset is pore width of ZSM‐5. m) Raman spectrum. Reproduced with permission.^[^ ^77^ ^]^ Copyright 2022, Wiley‐VCH.

Considering the higher electrochemical reactivity of water molecules in the solvation structure, numerous efforts have been dedicated to reducing bound water content at the metal/electrolyte interface through modifying the native hydration structure.^[^ ^17^ ^]^ The most straightforward approach involves minimizing direct contact between reactive water molecules and the Zn anode. Materials with porous structures, represented by metal‐organic frameworks (MOFs), covalent organic frameworks (COFs), and zeolitic imidazolate frameworks (ZIFs), have been reported to shield the attack of water molecules on metal electrodes based on the pore domain‐limiting effect.^[^ ^68^, ^69^, ^70^ ^]^ The microporous structure of MOFs promotes the passage of Zn^2+^ ions (1.86 Å) while hinders larger anions and water molecules, pre‐modulating the cation solvation structure before the electrode reaction. Functionalizing MOFs further enhances Zn^2+^ desolvation, enabling rapid ion migration and improving the performance of AZIBs. He et al. loaded functionalized MOF particles (NM‐125) on the commercial GF separator by in situ hydrothermal treatment (**Figure** **5**a).^[^ ^71^ ^]^ The smaller pore sizes of NM‐125 effectively prevent the passage of hydrated Zn^2+^ ions and anions, controlling water molecule‐induced side reactions. The ‐NH_2_ groups in NM‐125 attract Zn^2+^ ions and separate it from the solvated structure, facilitating rapid ion migration.

As illustrated in Figure 5b, Ma et al. further reduced separator thickness by loading UiO‐66‐NH_2_, a Zr‐based MOF, onto a 20 μm‐thick lignocellulosic separator.^[^ ^72^ ^]^ This particular MOF has subnanometer‐sized channels oriented internally along the [111] direction that can act as desolvation sieves, forcing cations to shed bound water molecules before reaching the electrode/electrolyte interface. The abundance of polar groups in the structure of UiO‐66‐NH_2_ is able to interact strongly with Zn^2+^, homogenizing the ion flux and immobilizing H_2_O molecules, and thereby improving electrochemical reversibility during long‐term cycling. Similarly, Li et al. synthesized a Cu‐based MOF (PFC‐31) and loaded it onto the GF separator using a pumping method (Figure 5c).^[^ ^73^ ^]^ In PFC‐31, the *π–π* interaction force between benzene rings (with a spacing of 3.199 Å) prevents the passage of [Zn(H_2_O)_6_]^2+^ through the separator to a certain extent, thus impeding the decomposition of water molecules originated from solvation structure on the anode. The results of Tafel and linear scanning voltammetry test show that the introduction of PFC‐31 significantly reduces the corrosion current density of symmetric cells and slowes down the onset of HER, implying a favorable effect of bound water limitation in eliminating side reactions (Figure 5d,e). Notably, the 1D structure of the specific MOF was able to provide a stable channel for ion transport, which contributes to promoting smooth Zn deposition.

In dilute zinc sulfate solutions, Zn^2+^ tends to exist in a hexahydrated structure (solvent‐separated ion pair, SSIP). However, when the concentration of zinc salt in solution is increased, more anions tend to enter the solvated sheath and undergo direct coordination with cations (contact ion pair, CIP).^[^ ^7^, ^74^, ^75^ ^]^ However, the high cost and viscosity of highly concentrated electrolytes limit their application, raising concerns about the introduction of numerous exotic additives. As a solution, the modification of the separator to force the electrolyte solvation structure transformation becomes an efficient strategy. Motivated by the efficient domain‐limiting effect brought about by the uniform pores of MOFs, Yang et al. used the classical microporous material, namely zeolite molecular sieves, which was ground into powder and mixed with polytetrafluoroethylene (PTFE) binder to form a freestanding flexible membrane as an effective electrolyte modification scheme.^[^ ^76^ ^]^ Zeolite molecular sieves offer advantages in terms of production cost and sustainability compared with other porous structures such as MOF. Upon exposure to the electrolyte, zeolite molecular sieves with a pore size of 3 Å tend to tightly adsorb water molecules within their pores and bind to the surface of the zeolite through spatial confinement and complex clustering of water molecules (Figure 5g). As shown in Figure 5h,i, COMSOL simulations combined with Faraday's law calculated the hydrogen precipitation during deposition, revealing the corrosion resistance in different systems. Compared to the unmodified system (HER rate 2.01 μL cycle^−1^), the HER rate (<0.1 μL cycle^−1^) in the zeolite‐modified electrolyte is significantly reduced, thereby enhancing Zn anode stability. Similarly, Zhu et al. used a molecular sieve electrolyte membrane with a channel size of 0.3–2.5 nm to limit the activity of both free and solvated water (Figure 5k,l).^[^ ^77^ ^]^ Raman spectroscopy revealed that this modification transformed the cation solvation structure from SSIP to CIP, accompanied by a significant decrease in the signaling response to free water, which implies that the domain‐limiting effect of the ZSM membrane managed to minimize the side‐reactions of the Zn anode (Figure 5m).

It is noteworthy that the disordered and excessively large pore structure of commercial GF separators hinders their effective regulation of metal cation behavior, while overly dense separators may adversely affect ion migration within the bulk phase. Consequently, systematic research on separator porosity remains essential to balance migration barriers with desolvation assistance. Given Zn metal exhibits exceptionally high Young's modulus, the mechanical properties of the separator emerge as a critical factor in suppressing dendrite penetration.

### 4.3 Water Environment Design

Apart from the bound water, the aqueous electrolyte in direct contact with the separator contains a large amount of free water, facilitating the fast transport of protons based on the Grotthuss transport mechanism.^[^ ^78^, ^79^ ^]^ The high electronegativity of the oxygen atom in water molecules induces a significant dipole moment in the hydrogen atoms, facilitating the formation of a continuous network of free water molecules that is mediated by hydrogen bonds within the bulk.^[^ ^80^ ^]^ In accordance with the Grotthuss mechanism, protons, driven by an external electric field, migrate rapidly toward the anode interface through a chain‐like transfer along the hydrogen bond network. The continuous accumulation of protons at the interface results in their reduction to hydrogen gas or their reaction with Zn^2+^ to form soluble zincates. These processes not only accelerate the depletion of the electrolyte but also cause fluctuations in the pH of the electrode surface and lead to the deposition of insulating by‐products. Consequently, these effects reduce the coulombic efficiency and shorten the cycle life of the cell. In this context, introducing organic functional groups is expected to offer hydrogen bonding sites, thereby facilitating the binding of water molecules and the reconfiguration of the bulk‐phase hydrogen bonding network.^[^ ^81^, ^82^, ^83^ ^]^ This approach aims to convert free water into a constrained state, thereby inhibiting the activity of water molecules.

**Figure 6:**
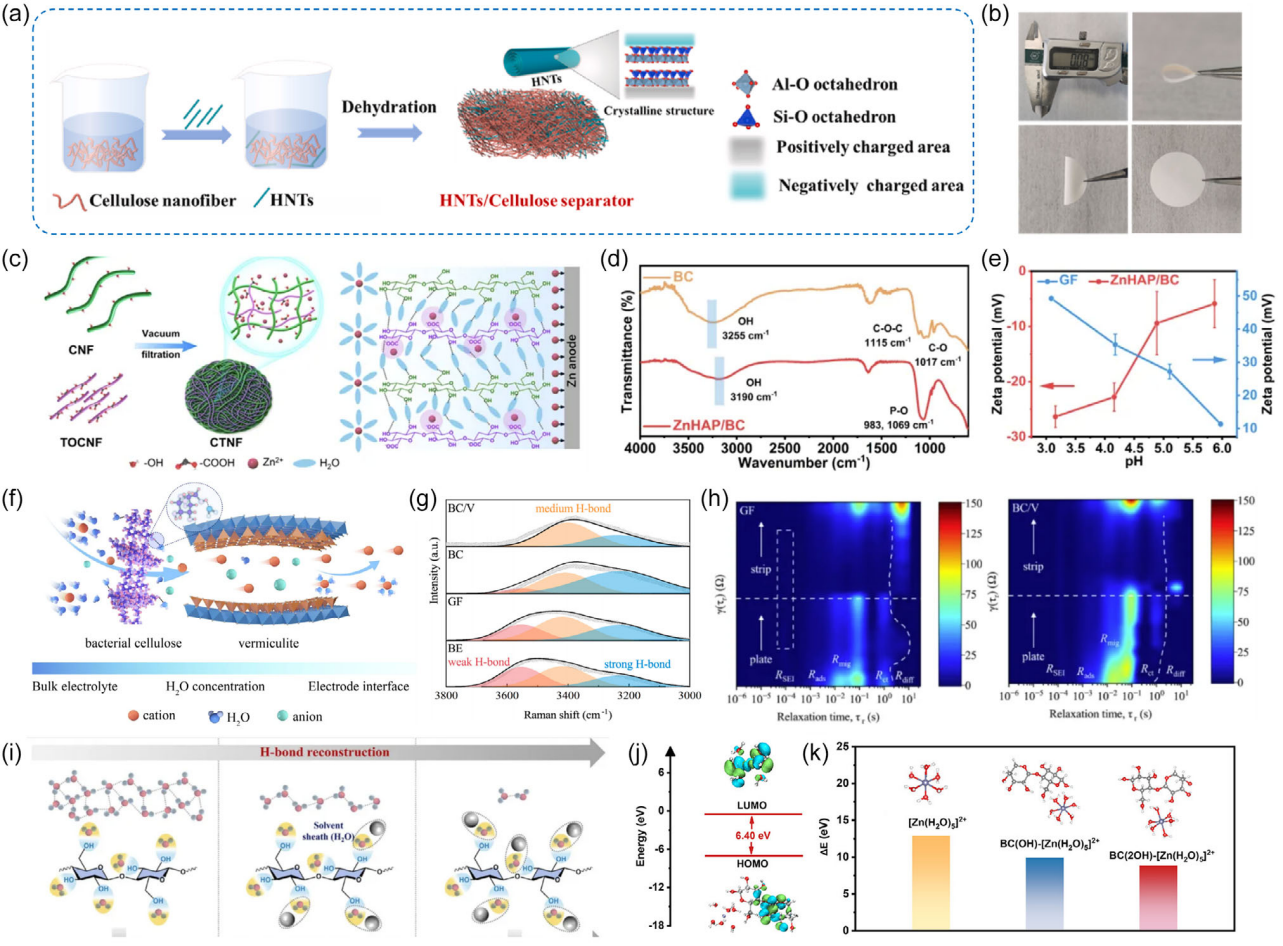
a) Synthesis steps of the HC separator. b) The digital photos of the modified separator. Reproduced with permission.^[^ ^84^ ^]^ Copyright 2025, Elsevier. c) Schematic illustration of synergistic mechanism of functional groups within the CTNF separator. Reproduced with permission.^[^ ^86^ ^]^ Copyright 2025, Elsevier. d) Attenuated Total Reflection Fourier Transform Infrared spectra of BC and ZnHAP/BC separator and e) Zeta potential test. Reproduced with permission.^[^ ^87^ ^]^ Copyright 2023, Elsevier. f) Regulatory mechanism of BC/V separator on the Zn^2+^ migration. g) Raman spectrum of electrolyte within different separator. h) In situ EIS results. Reproduced with permission.^[^ ^88^ ^]^ Copyright 2025, Elsevier. i) Mechanistic schematics of hydrophobicity gradient‐mediate separator. j) Energy levels of BC‐[Zn(H_2_O)_6_]^2+^. k) Energy barriers required for desolvation process of hydrated Zn^2+^. Reproduced with permission.^[^ ^89^ ^]^ Copyright 2025, Wiley‐VCH.

As shown in **Figure** **6**a, Wang et al. incorporated ≈50 wt% kaolin nanotubes (HNTs) into cellulose and fabricated composite cellulose separators (HCs) by solid solution casting. Compared with the inhomogeneous and irregular pores of GF, the HC separators exhibited excellent flexibility and smooth and dense surfaces, accompanied by the uniform dispersion of HNTs in their matrix (Figure 6b).^[^ ^84^ ^]^ The unique 1D tubular structure of HNTs, along with their charged cavities, is expected to accelerate the desolvation of Zn^2+^. Moreover, the HC demonstrated excellent water molecule adsorption capacity, which is favorable to mitigate the corrosion and damage to metals as caused by H_2_O cleavage on the electrode surface. Similarly, in combination with physical mixing and dehydration processes, the nano‐organic material lithium magnesium silicate (LMS) has also been reported to be introduced as a filler in separators made of cellulose nanofibers.^[^ ^85^ ^]^ Owing to the unique 3D spatial chain/layer structure and superior H_2_O adsorption capacity of LMS, the activation energy for desolvation of cations was significantly reduced and thereby inhibited water decomposition at the interface.

Yang et al. constructed all‐cellulose separators by integrating hydroxyl‐rich CNF and TEMPO oxidized cellulose nanofibers (TOCNF) containing abundant carboxyl groups (Figure 6c).^[^ ^86^ ^]^ Based on the synergistic interaction between hydroxyl and carboxyl groups in the cellulose structure, a unique Zn/hydrophilic network was established to limit water molecule activity through electrostatic coordination of the carboxyl groups to Zn^2+^ by electrostatic interactions and through hydroxyl‐dominated hydrogen bonding. In both hybrid supercapacitor and Zn//MnO_2_ battery systems, the CTNF diaphragm‐based full cells exhibit excellent multiplicity performance and operational stability. Qin et al. developed an environmentally friendly, biodegradable separator by integrating naturally derived bacterial cellulose (BC) with nano‐hydroxyapatite (HAP).^[^ ^87^ ^]^ This separator reduces water activity by forming hydrogen bonds between interfacial water molecules and the abundant polar functional groups in BC, thereby effectively passivating the electrode‐electrolyte interface and preventing parasitic reactions (Figure 6d). Simultaneously, the negatively charged surface separator promotes preferential migration of Zn^2+^ while electrostatically repelling SO_4_^2‐^ in the conduction channel, thereby greatly inhibiting the formation of by‐products at the interface (Figure 6e). Inspired by the enzyme‐gated ion channels in cell membranes, Li et al. designed a biomimetic separator prepared by introducing vermiculite into the BC substrate (Figure 6f).^[^ ^88^ ^]^ Layered vermiculite with negative surface charge distribution acted as a cation‐selective gate to enhance the selective transport and desolvation of cations. Meanwhile, the abundant hydroxyl groups on the modified separator allowed them to bond with water molecules through hydrogen bonding for the management of the electrolyte environment. According to Figure 6g, the Raman spectra further revealed that the interaction between water and the separator. The O—H stretching vibrations in the range of 3000–4000 cm^−1^ can be categorized into three characteristic peaks corresponding to strong, moderate and weak hydrogen bonding. Compared with the baseline electrolyte, the GF separators show negligible changes in hydrogen bond, indicating the minimal effect on the solvated structure of the electrolyte. The hydroxyl groups in the separator matrix and the Si—O and Mg—O bonds in the vermiculite disrupt the intrinsic hydrogen bonding network consisting of free water, thus forcing the anions to participate in the solvated structure. Furthermore, the results of the in situ electrochemical impedance spectroscopy (EIS) tests indicate that for GF separators, the 2D Zn deposition and solvation structure change inconsistently under the alternating field, accompanied by inhomogeneous deposition/exfoliation and bubble formation/fragmentation (Figure 6h). In contrast, the BC/V separator maintains a stable relaxation time under the alternating field, corresponding to the occurrence of smooth deposition. When composited with cellulose and BC, the BC substrate is rich in oxygen‐containing functional groups such as hydroxyl groups, which are capable of forming hydrogen bonds with water molecules in the solvation sheath and reorganizing them to inhibit water‐related side reactions on the electrode surface. Meanwhile, the HAP particles loaded on the BC fibers can promote the transport kinetics of Zn^2+^, thus equalizing the ion flux on the electrode, mitigating the occurrence of concentration polarization, and avoiding the longitudinal dendrite growth.

Considering that hydrophobic gradient structures exhibit unique control over hydrogen bonding network management, Dong et al. designed a negatively charged separator with a hydrophobic gradient structure by controlling the distribution of hydrophobic fumed silica (SiO_2_) on a BC substrate via impregnation (Figure 6i).^[^ ^89^ ^]^ In the hydrophilic BC region, the free water is converted to bound water by hydrogen bonding constraints, and the solvation structure of the cation initially removes part of the bound water. The decrease in the number of surrounding water molecules as Zn^2+^ migrates into the hydrophobic region containing more SiO_2_ results in the weakened interactions between anions and water, leading to the successive separation of the solvation sheath until the final completion of the desolvation process. Density‐functional theory (DFT) calculations showed that the oxygen‐containing groups on the BC surface significantly reduced the desolvation energy barrier of [Zn(H_2_O)_6_]^2+^ through the formation of strong hydrogen bonding, while the BC‐H_2_O bonding limited the electrochemical activity of water (Figure 6j,k). What's more, the negatively charged separator produces a selective electrostatic shielding effect, which specifically repels Cl^−^ in the seawater‐based electrolyte while facilitates Zn^2+^ transport, thus alleviating corrosion at the electrode/electrolyte interface.

Based on the above discussion, it is necessary to rationally design separators by considering factors such as binding energy, hydrophilicity, and hydrophobicity. Materials exhibiting high binding energy with water tend to disrupt the original hydrogen bond network or confine water molecules within the separator, thereby mitigating the decomposition reaction of active water molecules at the interface. Considering that the strength of hydrogen bonding varies depending on the dominant polar functional groups such as hydroxyl groups, establishing relevant descriptors or screening criteria can provide more effective theoretical guidance for suppressing HER and extending cycle life.

### 4.4 Deposition Orientation Optimization

Disordered Zn deposition leads to partial detachment of the deposited Zn from the electrode, which often induces coulombic efficiency reduction and side reaction occurrence, restricting the electrochemical performance of the anode.^[^ ^8^, ^90^ ^]^ Regulating the deposition morphology of Zn metal to realize the dense arrangement of Zn metal is of great significance to improve the cycle life.

**Figure 7:**
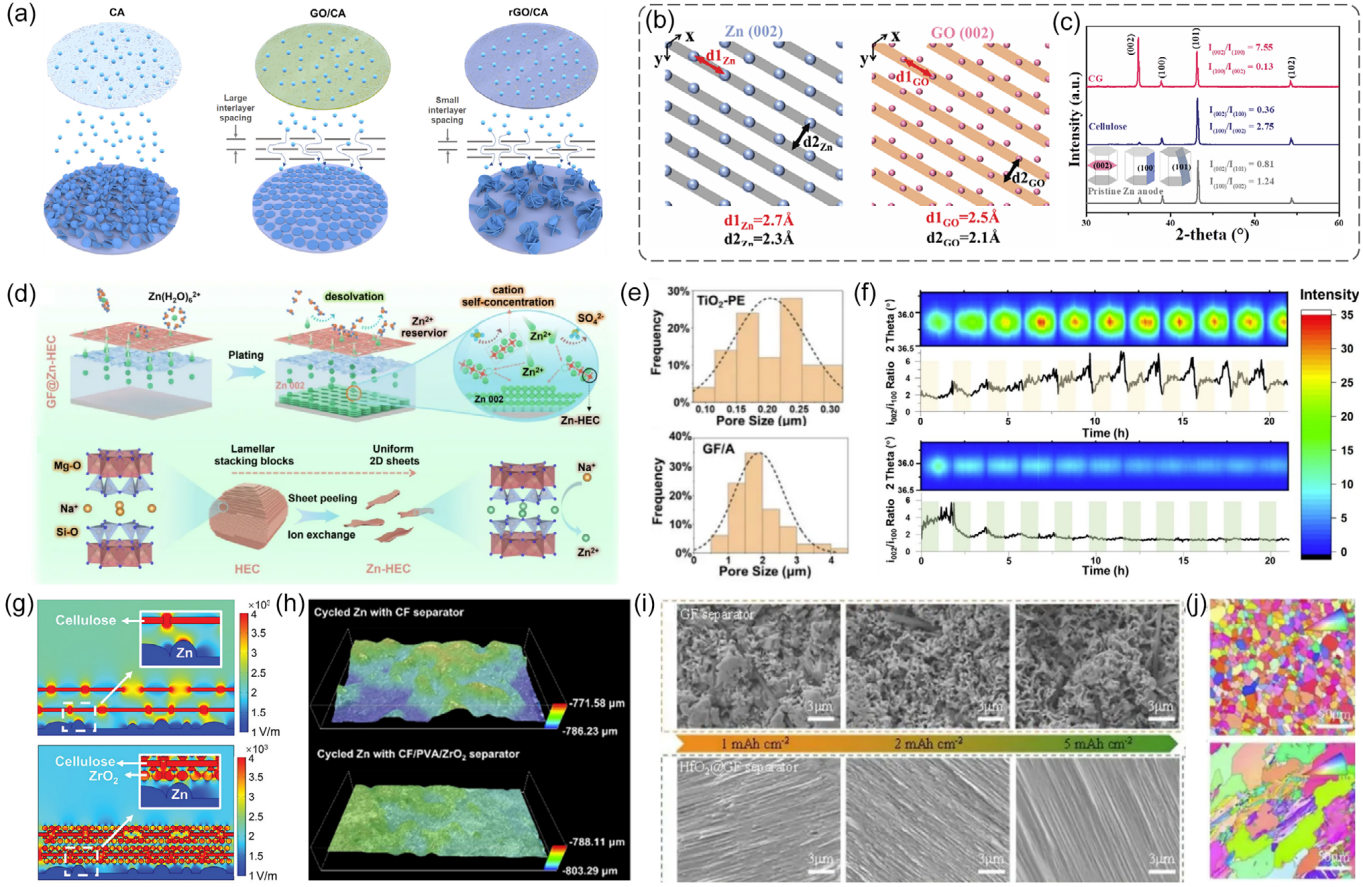
a) Scheme depicting the ion migration process through three types of separators. Reproduced with permission.^[^ ^99^ ^]^ Copyright 2023, American Chemical Society. b) Atomic arrangements of the (002) plane of Zn and GO, respectively. c) XRD patterns of cycled electrode. Reproduced with permission.^[^ ^100^ ^]^ Copyright 2021, Wiley‐VCH. d) Scheme illustrating Zn‐HEC inhibits side reactions. Reproduced with permission.^[^ ^101^ ^]^ Copyright 2025, Wiley‐VCH. e) The pore size distribution of the separators. f) The variation of (002) signal intensity and the relative ratio of the deposited Zn. Reproduced with permission.^[^ ^102^ ^]^ Copyright 2024, Elsevier. g) Electric field distribution result adjacent to the Zn anode. h) 3D depth‐enhanced microscopic images of the Zn electrode after certain cycles. Reproduced with permission.^[^ ^103^ ^]^ Copyright 2025, Wiley‐VCH. i) SEM images of the deposited Zn upon Cu foil at a current density of 1 mA cm^−2^. j) EBSD plots of Zn anodes after 50 cycles. Reproduced with permission.^[^ ^107^ ^]^ Copyright 2025, Wiley‐VCH.

In the case that the crystal structure of deposition is mainly with Zn(100), the deposited Zn will evolve into dendrites due to the large angle between the deposited metal and the substrate. In contrast, deposited Zn with (002) planes tend to be parallel to the surface.^[^ ^91^, ^92^ ^]^ In contrast, the Zn(002) provides a more stable surface, which is parallel to the electrode and possesses a high atomic packing density, low surface energy, and reduced electrochemical reactivity, mitigating corrosion and hydrogen precipitation.^[^ ^93^, ^94^ ^]^ The lattice mismatch between graphene materials and Zn(002) is only 7.4%, which contributes to the reduction of lattice strain during Zn deposition.^[^ ^95^, ^96^, ^97^ ^]^ On this basis, a functional septum consisting of graphene oxide (GO) with a separator substrate is capable of realizing uniformly oriented hexagonal (002) plane.^[^ ^98^ ^]^ To further simplify the preparation process, Luo et al. loaded GO onto the cellulose acetate (CA) surface by filtration, where the loading of GO was only 4 μg cm^−2^ (**Figure** **7**a).^[^ ^99^ ^]^ Unlike unmodified CA separators, the introduction of GO molecules led to the fact that Zn^2+^ could diffuse rapidly along defects, grain boundaries, and edge planes. The larger interlayer distance allows for larger transport channels for the ionic substances compared to the CA and rGO systems, and the GO membrane exhibits the highest diffusion coefficient. Meanwhile, due to the reduced content of O‐containing functional groups, the nucleation sites available in the rGO/CA separator are restricted and can hardly maintain the modulation of deposition. During cycling, the GO on the separator stays in direct contact with the Zn anode, guiding the growth of Zn deposition along a direction parallel to the (002) plane. Similarly, Cao et al. prepared composites of cellulose nanofibers and GO to spontaneously form self‐assembled films, which created good ionic conductivity through non‐valent bonding forces.^[^ ^100^ ^]^ During the initial stage of metal deposition, the low lattice mismatch between GO and Zn loaded on the fibers can induce preferential deposition of cations along the (002) plane (Figure 7b). Subsequently, these deposits further induce Zn deposition to grow in the same preferential direction, resulting in dendrite‐free Zn anodes. As shown in Figure 7c, the interstitial XRD signals of the electrode after cycling confirm the successful induction of Zn (002) planes by the synergistic effect of GO and cellulose.

In addition to the lattice‐matching based mechanism, the shielding effect of the separator on specific crystalline surfaces also contributes to the improvement of the deposition behavior. Huo et al. integrated layered zincized hectorite (Zn‐HEC) into GF, maintaining a nanoscale layered morphology well aligned with the HEC crystal structure (Figure 7d).^[^ ^101^ ^]^ In the unmodified system, Zn^2+^ exhibit similar binding energies in each crystal face of Zn, resulting in random deposition across multiple facets. Upon introducing the HEC modification, the binding energy between Zn^2+^ and Zn‐HEC with the Zn(002) plane decreases, while the binding energy with other crystal planes increases, which promotes preferential exposure of the Zn(002) plane during Zn deposition. These findings demonstrate that Zn‐HEC induces directional and homogeneous deposition, enabling symmetric cells to achieve an impressive cycle life of 2000 h at a high current density of 20 mA cm^−2^, with the modified separator that provides effective protection. In comparison to GF separators, which have larger pores, polyethylene (PE) membranes exhibit smaller and more uniformly distributed pore sizes. When the intrinsic pore size of the separator is smaller than the critical length of the crystals, Zn deposition tends to occur on the separator surface rather than within its pores. Yu et al. induced a deposition behavior along the horizontal direction using TiO_2_‐modified PE separators, whereas pores up to 2 μm in the GF led to randomly oriented growth of Zn metal (Figure 7e).^[^ ^102^ ^]^ The deposition/dissolution behavior of Zn metal at the interface of this novel separator was studied in combination with in situ X‐ray diffraction. As shown by the intensity of the Zn(002) signal and the relative ratio of crystalline surfaces, the cells with TiO_2_‐PE separator corresponded to a significant increase in the ratio of (002) crystalline plane, while the GF corresponded to the out‐of‐surface growth of metal Zn on the electrode surface oriented along the (100) and (101) surfaces (Figure 7f).

The optimization of the electric field, particularly through high‐dielectric constant materials, also plays a crucial role in regulating Zn deposition orientation. A functionalized separator was prepared by combining cellulose fibers (CF), ZrO_2_ and polyvinyl alcohol (PVA) by Zheng et al.^[^ ^103^ ^]^ The cellulose fiber and PVA together provided a certain mechanical strength to maintain the stability of the separator, while ZrO_2_ with a high dielectric constant regulated the local electric field. In general, electron accumulation at sites with high curvature in cellulose separators leads to a localized electric field enhancement, guiding Zn^2+^ reduction at protrusions during prolonged or repeated deposition, and ultimately promoting dendrite formation. In contrast, the introduction of ZrO_2_ nanoparticles homogenizes the electric field distribution, enhancing the exposure of the Zn(002) crystal plane and facilitating smoother Zn deposition (Figure 7g). As shown in Figure 7h, the electrode surface protected by the composite separator remains smooth and even, whereas the surface of a Zn electrode in contact with a CF separator exhibits irregular bumps. Notably, the (002) plane of Zn metal typically exhibits weak interactions with deposited atoms, hindering continuous planar epitaxy. In this case, other crystal planes including Zn(101) and Zn(100), with their higher surface energy and minimal lattice distortion, are conducive to smooth deposition.^[^ ^19^, ^104^, ^105^, ^106^ ^]^ The introduction of high‐dielectric constant materials like HfO_2_ into GF separators optimizes the polarized electric field at the interface, leading to improved Zn deposition behavior.^[^ ^107^ ^]^ Electron backscattering diffraction (EBSD) characterizes the crystallographic orientation information of the Zn anode. The Zn anode after cycling with GF separators displayed a disordered deposition behavior. In contrast, the HfO_2_@GF separator shows a predominant distribution dominated by green colored blocks representing the (101) crystallographic plane of the Zn metal, implying that HfO_2_ is conducive to promoting uniform plating/stripping behavior. As a result, symmetric cell assembled with the HfO_2_@GF separator was demonstrated for 4660 h at a current density of 5/1 mAh cm^−2^.

To date, the exploration of preferred crystal planes holds significant importance, as the dendrite growth issues they address play a decisive role in battery performance. As mentioned, direct contact between the separator and electrodes makes their modification or restructuring a favorable approach for regulating metal deposition behavior. Therefore, further investigation into the molecular polarity, adsorption energy toward different crystal planes, and charging properties of modifying materials or substrates remains warranted. Deriving systematic descriptors from this research to guide separator design is essential.

### 4.5 Practical Separator Design

The ideal separator material should possess several key performance indexes, including lightweight, porosity, ultra‐thinness, and high robustness.^[^ ^108^ ^]^ Among these, the optimization of separator thickness, as a core parameter directly affecting the volumetric energy density and ion transfer efficiency of the battery, is particularly critical. Currently, the thickness of widely used GF separators generally exceeds 50 μm, which seriously restricts the energy density improvement and industrialization of AZIBs.^[^ ^109^ ^]^ Despite efforts to reduce separator thickness through material optimization and process improvements, challenges remain in developing ultra‐thin separators. Reducing thickness often compromises mechanical strength and may also impairs electrolyte wettability and interfacial stability. Moreover, large‐scale production of ultra‐thin separators introduces significant quality control challenges.

**Figure 8:**
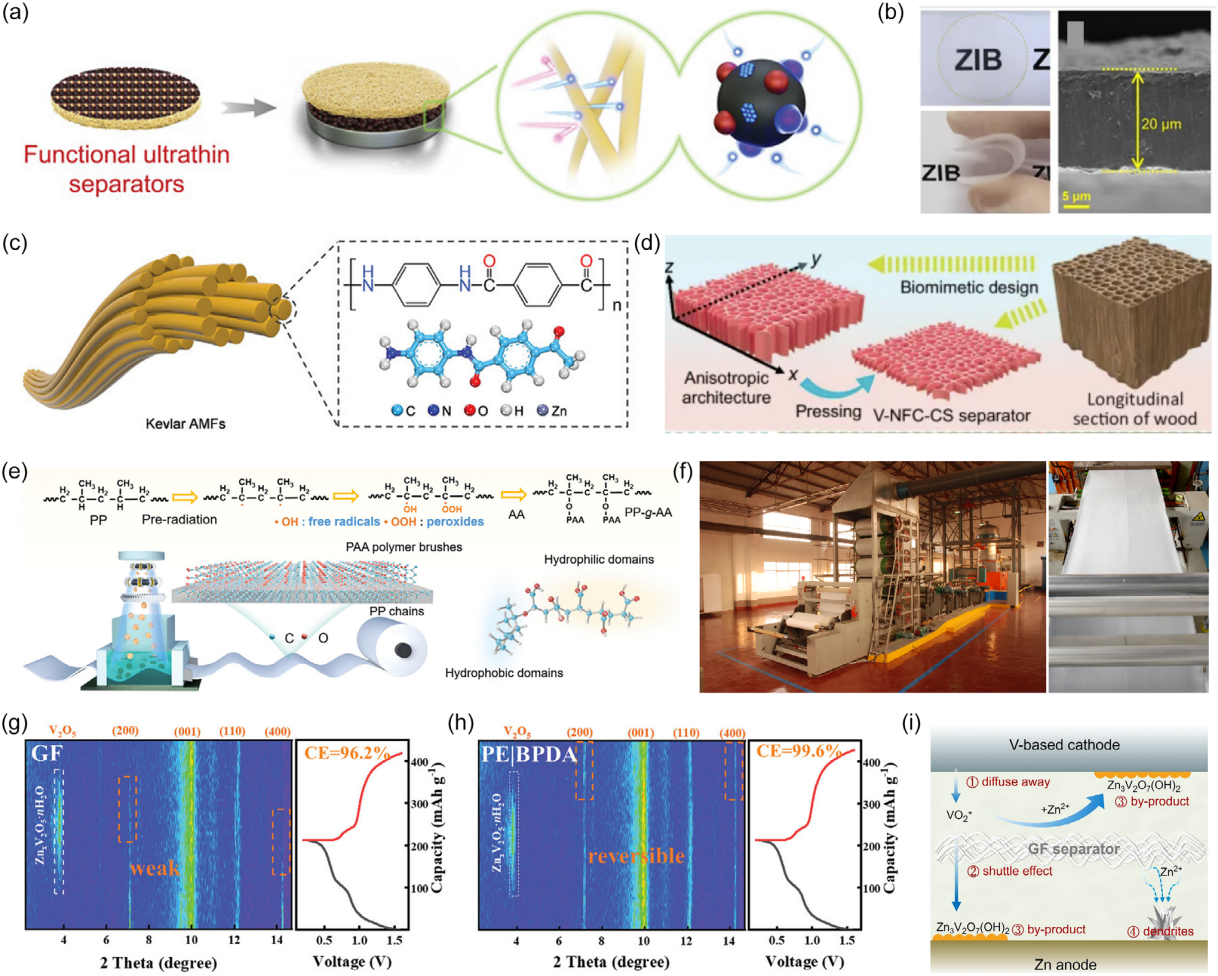
a) Fabrication schematics of the ultrathin separator. b) Photographs of the UCNF membrane. Reproduced with permission.^[^ ^110^ ^]^ Copyright 2023, Wiley‐VCH. c) The deprotonation process for preparing ANFs. Reproduced with permission.^[^ ^111^ ^]^ Copyright 2024, Wiley‐VCH. d) The generation of V‐NFC‐CS separator. Reproduced with permission.^[^ ^114^ ^]^ Copyright 2025, Springer Nature. e) Schematic illustration of the PP‐g‐AA separator generation. f) Large‐scale production. Reproduced with permission.^[^ ^115^ ^]^ Copyright 2024, Wiley‐VCH. g,h) The transmission‐mode Operando XRD pattern of the V_2_O_5_ cathode. Reproduced with permission.^[^ ^121^ ^]^ Copyright 2024, Wiley‐VCH. i) Diagram of the challenges for Zn/V cells. Reproduced with permission.^[^ ^122^ ^]^ Copyright 2024, Wiley‐VCH.

Li et al. designed a bilayer functional separator using an ultrathin carbon nanofiber (UCNF) membrane as a substrate and a MOF‐derived carbon‐coated copper (C/Cu) nanocomposite modified on its surface (**Figure** **8**a).^[^ ^110^ ^]^ As shown in Figure 8b, an ultrathin separator with a thickness of only 20 μm, accompanied by desirable mechanical strength and flexibility characteristics, was successfully prepared by a vacuum filtration process (Figure 8b). The heteroatom‐doped interface exhibited excellent zinc‐friendly properties, optimizing the Zn deposition interface and significantly enhancing electrode cycle life. Further, Yang et al. designed an aramid nanofiber (ANF) separator with abundant polar functional groups and interconnected nanopores (Figure 8c).^[^ ^111^ ^]^ The separator, reduced to 5 μm in thickness, maintained adequate mechanical strength. Meanwhile, the intrinsic nucleophilic carbonyl groups and uniform pore structure in the ANF fibers contribute to the regulation on the Zn^2+^ ion flux and electric field distribution on the surface of the Zn metal. On this basis, the symmetric cell corresponding to the ANF separator operated stably for more than 200 h even under 80% depth of discharge conditions.

Given the Young's modulus of Zn metal (108 GPa), the mechanical properties of the separator emerge as a key performance indicator for inhibiting dendrite penetration.^[^ ^112^, ^113^ ^]^ However, current studies rarely focus on documentation anisotropic separators for batteries, especially lacking a systematic study of the effect of modulus in the orientation direction. Inspired by the multistage oriented structure of natural wood, Ma et al. prepared a kind of optimized separators with vertically ordered aligned structures based on freeze‐drying technology (Figure 8d).^[^ ^114^ ^]^ Such a separator consists of nanofibrillated cellulose (NFC) and chitosan (CS), and the higher modulus endowed the separator with better mechanical properties, which was achieved to maintain its resistance to dendritic threats while the thickness was reduced. In addition, the V‐NFC‐CS separator introduces anisotropic channels in the electrolyte that are aligned along the z‐axis, helping to balance the conflict between high modulus and high ionic conductivity. In addition, in the process of commercialization of AZIBs, the high‐volume preparation of materials is an important factor in determining whether they can move from the laboratory to the mature production line. Zhu et al. successfully developed a polypropylene‐based separator (PP‐g‐AA) with an asymmetric molecular functional coating by radiation grafting (Figure 8e).^[^ ^115^ ^]^ This separator, only 10 μm thick with a mass of 0.68 mg cm^−^^2^, overcomes the limitations of conventional GF separators, which are typically too thick and heavy. The hydrophobic region formed by the polypropylene backbone effectively blocks the penetration of water molecules, while the grafted polyacrylic acid chain achieves a homogeneous distribution of ionic flux through the action of carboxyl functional groups. The lightweight and ultrathin nature of this separator effectively shortens the diffusion path of Zn^2+^ in the bulk phase, while increasing the bulk energy density of AZIBs. Surprisingly, the production scale of PP‐g‐AA separators can reach 10 000 m^2^ day^−1^ based on a rational production line design (Figure 8f).

Despite significant advancements in separator modification, current research predominantly focuses on regulating the metal/electrolyte interface at the anode side, with insufficient attention paid to the structure stability of the cathode materials and the shuttle effect of transition metal ions.^[^ ^116^, ^117^, ^118^ ^]^ In fact, as a key component that simultaneously contacts the electrodes, the separator is an ideal medium to realize synergistic regulation of both the anode and cathode interface.^[^ ^119^, ^120^ ^]^ Therefore, a coordinated synergistic optimization strategy for the positive and negative electrode interfaces can help to solve the performance bottleneck of existing modification methods in practical applications. Xue et al. introduced a functional layer of biphthalic anhydride (BPDA) on the positive side by ultrasonic spraying method to construct a selective ion transport barrier to reduce vanadium dissolution.^[^ ^121^ ^]^ Multi‐angle experiments such as in situ XRD in transmission mode and under long cycling conditions confirmed that the BPDA coating alleviated the self‐discharge phenomenon, while maintaining the structural stability of the cathode material (Figure 8 g). Meanwhile, the modification of bis(trifluoromethanesulfonyl)imide (BisSF) molecular layer on the anode side endowed the separator with an ion‐sieving function, inducing the Zn to be deposited along the (002) plane surface selectively. This bilaterally regulated design enabled the BPDA|PE|BisSF separator with a thickness of only 9 μm to exhibit excellent electrochemical performance in a soft‐packed battery assembled with matched V_2_O_5_ cathode, with volumetric and mass energy densities of 133.3 Wh L^−^^1^ and 71.4 Wh kg^−^^1^ and power outputs of 444.3 W L^−^^1^ and 238.0 W kg^−^^1^, respectively. Particularly, the separator design has good compatibility with the roll‐to‐roll manufacturing process and can be adapted for scale‐up production by optimizing the lamination parameters. Similarly, Qin et al. prepared polymer‐zeolite composite separators (SZ) by in situ growth of zeolite imidazolium backbone material (ZIF) on sodium alginate (SA) polymer‐based membranes (Figure 8i).^[^ ^122^ ^]^ The strong coordination formed between the carboxylic acid groups on the SA molecular chain and Zn^2+^ not only enhances the mechanical stability of the membrane material, but also provides the homogeneous growth of ZIF crystals. The abundance of nitrogen atoms in the SZ separator builds an effective ionic barrier through specific complexation with V^+^ on the one hand, significantly inhibiting the dissolution and shuttle effect of vanadium species on the anodic side, and realizing the redistribution of Zn^2+^ on the negative surface on the other hand, effectively inhibiting the growth of dendrites. In addition, to fit the intrinsically safe and environmentally friendly nature of AZIBs, the biodegradability and biocompatibility validation of the diaphragm are also worth incorporating into the comprehensive indexes of their evaluation. Ma et al. isolated a new biomass bamboo membrane (BM) as a separator from the lining of natural bamboo stems and utilized its inhibition of water molecule activity and anchoring to enhance the performance of the Zn anode in AZIBs.^[^ ^123^ ^]^ Compared with the conventional GF separator, the multistage 2D interlayer structure inside the BM separator endowed it with the function of regulating ion flux and promoting the uniform deposition. Most importantly, this is based on the fact that it was tested to start decomposing after 3 days in a natural environment and was almost completely decomposed by 30 days, confirming the excellent degradability and biocompatibility of separators made from natural biomass‐based materials. Li et al. developed a biodegradable lignocellulosic nanofiber (LCNF) separator derived from waste palm biomass.^[^ ^124^ ^]^ This material effectively modulates the solvation structure within the electrolyte solution, thereby mitigating water‐induced corrosion reactions. With uniformly distributed nanopores and a negatively charged surface, the separator promotes preferential deposition along the (002) crystal plane. Comparative soil degradation tests revealed that the LCNF separator completely decomposed within two weeks, while the GF separator maintained its original structure, demonstrating the wood‐based separator's advantage in optimizing post‐use disposal. Additionally, material recycling plays a crucial role in resource regeneration within battery technology and holds potential for enhancing the economic viability of AZIBs.^[^ ^125^, ^126^ ^]^ In this context, Yao et al. introduced a novel P/FS‐Z separator, developed using an innovative wet‐rolling process.^[^ ^127^ ^]^ This separator integrates a PTFE matrix with hydrophilic fumed silica (FSS) filler and zinc salts, achieving a precise balance between hydrophilic and hydrophobic properties. Notably, the P/FS‐Z separator can be fully recovered by rinsing with dilute oxalic acid, with reconstituted batteries exhibiting excellent capacity retention.

## 5 Conclusion and Future Outlooks

AZIBs are considered as promising candidates for grid‐level energy storage systems due to their advantages of intrinsic safety, economy, and high theoretical capacity. However, practical applications of AZIBs are significantly hindered by challenges such as dendrite growth, HER, corrosion, and dissolution of cathode materials. As a key component of the battery system, the separator shows great potential to solve these technical bottlenecks based on multiple mechanisms, such as regulating the ion transport behavior, inhibiting the occurrence of side reactions and guiding the uniform Zn deposition.

**Figure 9:**
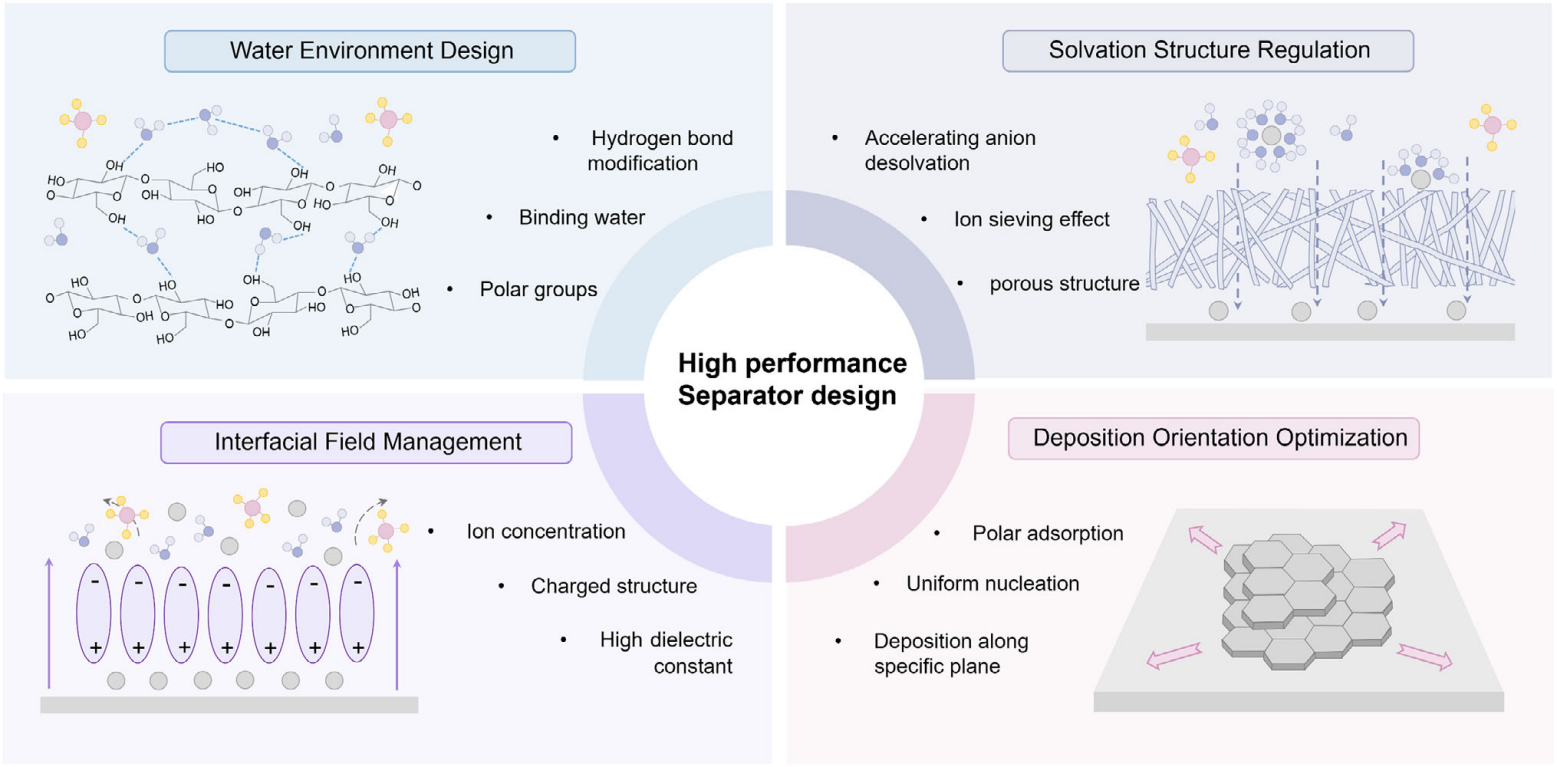
Scheme illustrating the working mechanism of functional separator for AZIBs.

The optimization of separators could achieve performance enhancement, which is mainly based on the following mechanisms (**Figure** **9**). Through building a uniform and dense pore structure, the new and modified GF separators are able to achieve uniform Zn^2+^ transport and effectively inhibit the formation of dendrites and the generation of “dead Zn.” This optimized pore structure also reduces the continuous accumulation of water molecules at the interface, avoiding the damage to the electrode caused by unfavorable side reactions. Second, the hydrogen bonding based on polar functional groups exerts a strong restraining effect on the catastrophic water molecules during the electrode reaction process, avoiding them from being in the process. Meanwhile, the application of functional materials with ion‐sieving properties can precisely regulate the interfacial ion concentration at the same time as the solvation structure of the ions to play an effective regulation. In addition, the modified materials with special polarization effect or with electric charge can evenly distribute the electric field and ion flux. Finally, the adsorption or shielding effect on specific deposition crystal surfaces enables the modified separators to achieve negative‐side oriented deposition, and the dense and uniform deposition is utilized to extend the cycle life of the cell. This article comprehensively summarizes the separator engineering strategies tailored for AZIBs with relevant discussions offered in the previous sections (**Table** **1**).

**Table 1:** Summary of the symmetric cell performances and working mechanism with modified separator.

Substrate	Modification material	Working mechanism	Lifespan Cycling condition	Ref.
Bacterial cellulose	Ag nanowires	Accelerating Zn^2+^ transport	1000 h (80 mA cm^−2^, 1 mA h cm^−2^)	[57]
Bacterial cellulose	BaTiO_3_	High dielectric constant	2880 h (1 mA cm^−2^, 0.05 mA h cm^−2^)	[58]
Nanocellulose	Zr^4+^‐hydrolysate	Modulating electric field	1600 h (1 mA cm^−2^, 1 mA h cm^−2^)	[61]
Glass fiber	BaTiO_3_	Regulating Zn^2+^ transport	1600 h (10 mA cm^−2^, 2.5 mA h cm^−2^)	[62]
Glass fiber	Mxene@NiO	Regulating Zn^2+^ transport	2400 h (2 mA cm^−2^, 2 mA h cm^−2^)	[56]
Glass fiber	Vertical graphene	Modulating electric field	300 h (0.5 mA cm^−2^, 0.5 mA h cm^−2^)	[39]
Bacterial cellulose	SiO_2_	Hydrophobicity gradient structure	1400 h (10 mA cm^−2^, 2.5 mA h cm^−2^)	[89]
Cellulose nanofibers	Lithium magnesium silicate	Water adsorption	1000 h (1 mA cm^−2^, 0.5 mA h cm^−2^)	[85]
Cellulose	Halloysite nanotubes	Electrostatic interaction	2700 h (1 mA cm^−2^, 0.5 mA h cm^−2^)	[84]
Cellulose nanofiber	TEMPO	Electrostatic interaction Water adsorption	1800 h (1 mA cm^−2^, 0.5 mA h cm^−2^)	[86]
Bacterial cellulose	Vermiculite	Regulating both Zn^2+^ and H_2_O transport	1500 h (DOD 20%) (1.169 mA cm^−2^, 1.169 mA h cm^−2^)	[88]
Lignocellulose	−NH_2_‐functionalized Zr‐based MOF	Facilitating desolvation	2000 h (2 mA cm^−2^, 2 mA h cm^−2^)	[72]
Glass fiber	MOF	Facilitating desolvation	1100 h (0.5 mA cm^−2^, 0.25 mA h cm^−2^)	[73]
/	Zeolite	Restricting solvated water	4765 h (0.8 mA cm^−2^, 0.8 mA h cm^−2^)	[76]
/	Zeolite	Restricting solvated water	2000 h (1 mA cm^−2^, 1 mA h cm^−2^)	[77]
Glass fiber	ZIF‐8	Facilitating desolvation	2300 h (0.5 mA cm^−2^, 0.25 mA h cm^−2^)	[128]
Cellulose acetate	Graphene oxide	Oriented deposition	500 h (10 mA cm^−2^, 1 mA h cm^−2^)	[99]
Glass fiber	HfO_2_	High dielectric constant Oriented deposition	4660 h (5 mA cm^−2^, 1 mA h cm^−2^)	[107]
Cellulose nanofiber	Graphene oxide	Electrostatic interaction Oriented deposition	1750 h (2 mA cm^−2^, 1 mA h cm^−2^)	[100]
Glass fiber	Zincized hectorite	Cation self‐concentration Oriented deposition	2000 h (20 mA cm^−2^, 5 mA h cm^−2^)	[101]
Glass fiber	Anatase TiO_2_	Crystalline structure engineering Oriented deposition	2000 h (2 mA cm^−2^, 2 mA h cm^−2^)	[129]
Polyethylene	TiO_2_	Oriented deposition	500 h (1 mA cm^−2^, 1 mA h cm^−2^)	[102]
Cellulose	ZrO_2_/polyvinyl alcohol	High dielectric constant Oriented deposition	1750 h (1 mA cm^−2^, 0.5 mA h cm^−2^)	[103]
Aramid nanofiber	/	Oriented deposition	850 h (5 mA cm^−2^, 2.5 mA h cm^−2^)	[111]
Polypropylene	Acrylic acid	Regulating Zn^2+^ transport Oriented deposition	1800 h (1 mA cm^−2^, 1 mA h cm^−2^)	[115]
Polyethylene	Anhydride/3,3′‐diamino‐4,4′‐dihydroxydiphenyl sulfone	Inhibiting vanadium dissolution and shuttling Regulating ion transport	500 h (5 mA cm^−2^, 5 mA h cm^−2^)	[121]
Cellulose nanofiber	C/Cu nanocomposite‐MOF	Optimizing deposition interface	2000 h (1 mA cm^−2^, 0.5 mA h cm^−2^)	[110]
Bamboo membrane	/	Binding water Regulating Zn^2+^ transport	1000 h (1 mA cm^−2^, 1 mA h cm^−2^)	[123]
Polymeric	Zeolite	Preventing vanadium shuttling Regulating Zn^2+^ transport	2500 h (1 mA cm^−2^, 1 mA h cm^−2^)	[122]
Poly tetrafluoroethylene	Fumed silica	Regulating Zn^2+^ transport Maintaining interface hydrophobicity	1200 h (20 mA cm^−2^, 10 mA h cm^−2^)	[127]
Nanofibrillated cellulose	Chitosan	Enhancing modulus Regulating Zn^2+^ transport	1000 h (10 mA cm^−2^, 2 mA h cm^−2^)	[114]
Mixed cellulose ester	/	Regulating Zn^2+^ transport	2700 h (1 mA cm^−2^, 1 mA h cm^−2^)	[109]
Sulfonated bacterial cellulose	/	Regulating Zn^2+^ transport	1200 h (1 mA cm^−2^, 1 mA h cm^−2^)	[42]
Lignocellulose nanofibers	Deep eutectic solvents	Facilitating desolvation Oriented deposition	3500 h (0.5 mA cm^−2^, 1 mA h cm^−2^)	[124]

However, current separator designs for zinc‐based energy storage devices still face numerous bottlenecks that limit their further advancement, including 1) performance trade‐offs: many current membrane modification strategies inherently involve performance trade‐offs, where optimizing one specific parameter often leads to the deterioration of other critical characteristics. For instance, enhancing mechanical strength typically compromises ionic conductivity, as denser structures impede ion transport pathways. Similarly, increasing separator thickness to prevent dendrite penetration significantly reduces the battery's volumetric energy density, creating a fundamental conflict with the design goals of high‐energy storage systems. This interdependence between performance metrics underscores the limitations of 1D optimization approaches and highlights the urgent need for multiscale, collaborative design to achieve a balanced development of separator properties. The integration of electrolyte and separator may represent a future development direction for separator design. Achieving precise control of the interfacial microenvironment through in‐situ polymerization to construct gel electrolyte/separator composite systems, or by designing smart separators with multifunctional layered structures that combine ion conductivity, electron insulation, and mechanical barrier properties. 2) Insufficient long‐term stability: Under prolonged cycling or high current density conditions, the functional protective layer of certain modified separators may undergo delamination or structural degradation, leading to a significant decline in protective effectiveness. Simultaneously, self‐supporting separators face critical challenges in aqueous electrolyte environments, including controlling swelling rates and ensuring hydrolysis stability. The long‐term integrity of their internal structure and sustained protective capability for electrodes remain difficult to guarantee under extreme electrochemical conditions. These issues related to both interface and bulk stability significantly constrain the large‐scale application of advanced separators in practical battery systems. In this sense, the introduction of easily hydrolyzed bonds should be avoided during separator design, with priority given to stable chemical bonds such as ether or amide linkages. Furthermore, employing in situ polymerization techniques like electrochemical polymerization or UV curing to form an interpenetrating network structure between the functional layer and base membrane helps prevent interlayer delamination and maintains the separator's long‐term operational stability. 3) Practical application challenges: Despite the outstanding properties exhibited by various functional materials in current research, their synthesis processes often involve complex reaction pathways and stringent preparation conditions (e.g., high temperature and pressure, inert atmosphere), which severely limit their practical feasibility. Additionally, while certain separator fabrication techniques achieve ideal structural control at the laboratory scale, they fail to meet the dual demands of industrialization—economic viability and large‐scale production stability—due to factors such as high raw material costs, low production efficiency, and technical bottlenecks that hinder scalability. Furthermore, current research predominantly focuses on laboratory‐scale electrochemical performance testing and lacks unified industrial application evaluation standards, making it difficult to assess their potential for practical applications accurately.

In order to prepare future‐proof high‐performance AZIBs, there are a number of key points to consider in the research of separator engineering: First is innovative advancement of characterization. Although there have been some studies focusing on the regulation of metal/electrolyte interface and bulk phase electrolyte by separators, among them, the mechanisms by which separators regulate microscopic processes, such as ion migration modes, formation of interfacial components, and induction of deposition orientation, are not yet clear. The resolution of these microscopic processes urgently requires the development of multiscale and multidimensional in situ characterization tests suitable for aqueous liquid‐phase environments. Particularly, it is critical to break through the temporal and spatial resolution limitations of existing characterization techniques. For example, synchrotron X‐ray tomography and high‐throughput in situ X‐ray diffraction have the potential to provide direct visual evidence of metal deposition/dissolution processes, especially for atomic‐scale observation of the dynamic evolution of interfaces. Furthermore, establishing a “characterization‐simulation” paradigm that combines experimental data with molecular dynamics simulations or finite element analysis is expected to uncover the fundamental mechanisms by which separators regulate microscopic processes, providing valuable theoretical insights for designing functional separators. Second is integrated design of separator mechanisms. During the process of electrode cycling, there is a possibility of shedding, failure, or degradation of the modifying materials, leading to difficulties in maintaining the long‐term stable efficacy of the separator. Meanwhile, the response of the separator itself that withstands deformation during repeated plating/stripping is also a serious challenge. Although extensive research has been conducted, there is still a need to systematically investigate more stable material loading processes, targeted functional group modifications, and effective regulation of porosity. For example, some of the surface termination groups can play a role in constraining water molecules and regulating local electric field strength and ionic fluxes, but they are not yet well understood. Based on the strength of hydrogen bonding interactions, the solvents selected during the preparation of cellulose separators have an important influence on the basic physicochemical properties of the separators. Therefore, a rational system of descriptors needs to be established to guide the screening of solvents, functional groups, and substrate materials and thus to elucidate the design guidelines for high‐performance separators applicable to AZIBs. It is worth noting that the optimized design of separators needs to comprehensively consider the synergistic and constraining relationships of key parameters such as electrochemical performance, mechanical strength and thickness to achieve overall performance improvement. Third is comprehensive cost–benefit considerations. The current development of separator modification technology still faces significant bottlenecks in industrialization. Due to the complex preparation process, the maximum preparable area and average production cycle of most separator modification strategies are still at the laboratory level, which is difficult to meet the needs of industrialized production. In order to advance practical applications, there is an urgent need to develop processes such as continuous roll‐to‐roll production, low‐energy drying technologies, and material loading methods with high raw material utilization. Although the cellulose, polypropylene, polytetrafluoroethylene, and other polymer‐based composite separators are particularly noteworthy in recent years, which exhibit promising application prospects, they are still in the early stages of exploration. Finally is the unified evaluation metrics development. There are still several key issues to be solved in the performance evaluation system of diaphragm materials. First, there is still a lack of a unified evaluation system for the physical parameters of diaphragms before and after modification (such as dissolution rate, porosity, etc.), which makes the direction of regulating the structure and physical properties of diaphragms unclear. Furthermore, despite the intrinsic safety advantages of aqueous batteries, organic components that may remain during organic solvent treatment or chemical modification can negatively affect the biocompatibility of separators, which is a potential risk since there is no standardized evaluation method established. In order to build zinc‐based energy storage devices for real‐world applications, separator engineering must face rigorous testing under extreme conditions and actual operating environments such as wide‐temperature domain stability and bending/puncture resistance. In addition, given the intrinsic high safety qualities of aqueous batteries, in addition to conventional electrochemical performance, multidimensional evaluation criteria such as biodegradation rate, toxicology testing, and fail‐safe validation for environmental friendliness need to be urgently developed. Such a comprehensive evaluation framework will provide important guidance for the design of next‐generation high‐performance separators.

## Conflict of Interest

The authors declare no conflict of interest.

## References

[1] M. I. Hoffert , K. Caldeira , G. Benford , D. R. Criswell , C. Green , H. Herzog , A. K. Jain , H. S. Kheshgi , K. S. Lackner , J. S. Lewis , H. D. Lightfoot , W. Manheimer , J. C. Mankins , M. E. Mauel , L. J. Perkins , M. E. Schlesinger , T. Volk , T. M. Wigley , Science 2002, 298, 981. 10.1126/science.1072357 12411695

[2] Q. Zhang , Y. Su , Z. Shi , X. Yang , J. Sun , Small 2022, 18, 2203583. 10.1002/smll.202203583 35996805

[3] Y. Su , X. Yang , Q. Zhang , J. Sun , Z. Liu , J. Electroanal. Chem. 2022, 904, 115883.

[4] F. Creutzig , P. Agoston , J. C. Goldschmidt , G. Luderer , G. Nemet , R. C. Pietzcker , Nat. Energy 2017, 2, 17140.

[5] J. B. Goodenough , Nat. Electron. 2018, 1, 204.

[6] D. Han , C. Cui , K. Zhang , Z. Wang , J. Gao , Y. Guo , Z. Zhang , S. Wu , L. Yin , Z. Weng , F. Kang , Q.‐H. Yang , Nat. Sustain. 2021, 5, 205.

[7] W. Guo , L. Xu , Y. Su , Z. Tian , C. Qiao , Y. Zou , Z. Chen , X. Yang , T. Cheng , J. Sun , ACS Nano 2024, 18, 10642. 38560784 10.1021/acsnano.4c02740

[8] P. Wang , S. Liang , C. Chen , X. Xie , J. Chen , Z. Liu , Y. Tang , B. Lu , J. Zhou , Adv. Mater. 2022, 34, e2202733. 35746854 10.1002/adma.202202733

[9] S. Lei , Z. Liu , C. Liu , J. Li , B. Lu , S. Liang , J. Zhou , Energy Environ. Sci. 2022, 15, 4911.

[10] L. E. Blanc , D. Kundu , L. F. Nazar , Joule 2020, 4, 771.

[11] M. Zhang , W. Xu , X. Han , H. Fan , T. Chen , Y. Yang , Y. Gao , C. Zheng , Y. Yang , T. Xiong , Y. W. Zhang , W. S. V. Lee , W. Wang , H. Pan , Z. G. Yu , J. Xue , Adv. Energy Mater. 2024, 14, 2303737.

[12] Z. Zhang , B. Xi , X. Ma , W. Chen , J. Feng , S. Xiong , SusMat. 2022, 2, 114.

[13] Z. Tian , W. Guo , Z. Shi , Z. Alhubail , Y. Wang , D. Alsulaiman , Y. Zhu , J. Ming , J. Sun , H. N. Alshareef , ACS Energy Lett. 2024, 9, 5179.

[14] T. Sun , Q. Nian , X. Ren , Z. Tao , Joule 2023, 7, 2700.

[15] Z. Zhou , M. Han , Y. Sun , Y. Cui , S. A. El‐khodary , D. H. L. Ng , J. Lian , J. Ma , Adv. Funct. Mater. 2023, 34, 2308834.

[16] T. Wang , P. Wang , L. Pan , Z. He , L. Dai , L. Wang , S. Liu , S. C. Jun , B. Lu , S. Liang , J. Zhou , Adv. Energy Mater. 2022, 13, 2203523.

[17] L. Geng , J. Meng , X. Wang , C. Han , K. Han , Z. Xiao , M. Huang , P. Xu , L. Zhang , L. Zhou , L. Mai , Angew. Chem. Int. Ed. 2022, 61, e202206717. 10.1002/anie.202206717 35610667

[18] W. Chen , S. Guo , L. Qin , L. Li , X. Cao , J. Zhou , Z. Luo , G. Fang , S. Liang , Adv. Funct. Mater. 2022, 32, 2112609.

[19] Y. Wang , L. e. Mo , X. Zhang , Y. Ren , T. Wei , Z. Li , Y. Huang , H. Zhang , G. Cao , L. Hu , Adv. Energy Mater. 2023, 13, 2301517.

[20] F. Wang , O. Borodin , T. Gao , X. Fan , W. Sun , F. Han , A. Faraone , J. A. Dura , K. Xu , C. Wang , Nat. Mater. 2018, 17, 543. 29662160 10.1038/s41563-018-0063-z

[21] Z. Zhao , R. Wang , C. Peng , W. Chen , T. Wu , B. Hu , W. Weng , Y. Yao , J. Zeng , Z. Chen , P. Liu , Y. Liu , G. Li , J. Guo , H. Lu , Z. Guo , Nat. Commun. 2021, 12, 6606. 34785684 10.1038/s41467-021-26947-9 PMC8595410

[22] L. Miao , Z. Xiao , D. Shi , M. Wu , D. Liu , Y. Li , X. Liu , Y. Sun , S. Zhong , Z. Qian , R. Wang , Adv. Funct. Mater. 2023, 33, 2306952.

[23] W. Guo , T. Hua , C. Qiao , Y. Zou , Y. Wang , J. Sun , Energy Storage Mater. 2024, 66, 103244.

[24] F. Sun , Q. Tang , D.‐e. Jiang , ACS Catal. 2022, 12, 8404.

[25] Y. Liang , M. Qiu , P. Sun , W. Mai , Adv. Funct. Mater. 2023, 33, 2304878.

[26] Q. Yang , Q. Li , Z. Liu , D. Wang , Y. Guo , X. Li , Y. Tang , H. Li , B. Dong , C. Zhi , Adv. Mater. 2020, 32, 2001854. 10.1002/adma.202001854 33103828

[27] J. Zheng , L. A. Archer , Sci. Adv. 2021, 7, eabe0219. 33523975 10.1126/sciadv.abe0219 PMC7787491

[28] W. Wang , R. Huang , Y. Tao , P. He , S. Tuo , Y. Bian , R. Hu , J. Yan , Y. Liang , W. Zhang , Rare Met. 2024, 43, 4992.

[29] Q. Yang , G. Liang , Y. Guo , Z. Liu , B. Yan , D. Wang , Z. Huang , X. Li , J. Fan , C. Zhi , Adv. Mater. 2019, 31, 1903778. 10.1002/adma.201903778 31517400

[30] W. Du , Z. Zhang , F. Iacoviello , S. Zhou , R. E. Owen , R. Jervis , D. J. L. Brett , P. R. Shearing , ACS Appl. Mater. Interfaces 2023, 15, 14196. 36892017 10.1021/acsami.2c19895 PMC10037236

[31] H. Liu , X. Jiang , Z. Hu , Z. Han , J. Chen , K. Bai , Y. Zhang , W. Du , M. Ye , Y. Tang , X. Liu , Z. Wen , C. C. Li , Adv. Mater. 2025, 10.1002/adma.202509622. 40552541

[32] Q. Nian , X. Luo , D. Ruan , Y. Li , B. Q. Xiong , Z. Cui , Z. Wang , Q. Dong , J. Fan , J. Jiang , J. Ma , Z. Ma , D. Wang , X. Ren , Nat. Commun. 2024, 15, 4303. 38773073 10.1038/s41467-024-48444-5 PMC11109197

[33] Y. Zhao , S. Guo , M. Chen , B. Lu , X. Zhang , S. Liang , J. Zhou , Nat. Commun. 2023, 14, 7080. 37925505 10.1038/s41467-023-42919-7 PMC10625522

[34] J. Yang , R. Zhao , Y. Wang , Z. Hu , Y. Wang , A. Zhang , C. Wu , Y. Bai , Adv. Funct. Mater. 2023, 33, 2213510.

[35] M. Chen , Y. Ma , N. Li , L. Li , D. Xiao , C. Zhi , Q. Chen , ACS Energy Lett. 2025, 10, 2440.

[36] W. Gu , K. Wu , J. Huang , X. Yang , X. Huang , Z. Dong , S. Shen , Y. Bai , H. K. Liu , S. X. Dou , C. Wu , Adv. Energy Mater. 2025, 10.1002/aenm.202502652.

[37] H. Du , Z. Yi , H. Li , W. Lv , N. Hu , X. Zhang , W. Chen , Z. Wei , F. Shen , H. He , Chemistry 2024, 30, e202303461. 38050714 10.1002/chem.202303461

[38] H. Wang , W. Ye , B. Yin , K. Wang , M. S. Riaz , B. B. Xie , Y. Zhong , Y. Hu , Angew. Chem. Int. Ed. 2023, 62, e202218872. 10.1002/anie.202218872 36647214

[39] C. Li , Z. Sun , T. Yang , L. Yu , N. Wei , Z. Tian , J. Cai , J. Lv , Y. Shao , M. H. Rummeli , J. Sun , Z. Liu , Adv. Mater. 2020, 32, e2003425. 32656930 10.1002/adma.202003425

[40] L. Cao , D. Li , T. Deng , Q. Li , C. Wang , Angew. Chem. Int. Ed. 2020, 59, 19292. 10.1002/anie.202008634 32638488

[41] Z. Zhang , Y. Li , X. Yin , S. Li , B. Li , N. Zhao , J. Zhu , L. Dai , L. Wang , Z. He , Z. Feng , Green Energy Environ. 2025, 10.1016/j.gee.2025.03.010.

[42] W. Yan , J. Xian , S. Huang , Y. Leng , Q. Liu , T. Xiao , Y. Zhao , P. Yang , Y. Wu , Energy Storage Mater. 2025, 76, 104150.

[43] X. Yang , Z. Zhang , M. Wu , Z. P. Guo , Z. J. Zheng , Adv. Mater. 2023, 35, 2303550. 10.1002/adma.202303550 37528474

[44] H. Zhang , X. Gan , Z. Song , J. Zhou , Angew. Chem., Int. Ed. 2023, 62, e202217833. 10.1002/anie.202217833 36720709

[45] Y. Wang , L. Xu , X. Chen , Z. Chen , X. Li , W. Guo , T. Cheng , Y. Yi , J. Sun , ACS Nano 2025, 19, 3920. 39813795 10.1021/acsnano.4c16664

[46] L. Cheng , W. Li , M. Li , S. Zhou , J. Yang , W. Ren , L. Chen , Y. Huang , S. Yu , J. Wei , Adv. Funct. Mater. 2024, 34, 2408863.

[47] N. Shen , S. Dai , G. Zhou , J. Miao , Z. Hu , G. Zhi , X. Li , H. Wang , D. Kong , T. Xu , Z. Zhang , X. Li , H. Y. Yang , Y. Wang , Adv. Funct. Mater. 2024, 35, 2417809.

[48] B. Wu , Y. Wu , Z. Lu , J. Zhang , N. Han , Y. Wang , X.‐m. Li , M. Lin , L. Zeng , J. Mater. Chem. A 2021, 9, 4734.

[49] Y. Cui , Q. Zhao , X. Wu , X. Chen , J. Yang , Y. Wang , R. Qin , S. Ding , Y. Song , J. Wu , K. Yang , Z. Wang , Z. Mei , Z. Song , H. Wu , Z. Jiang , G. Qian , L. Yang , F. Pan , Angew. Chem. Int. Ed. 2020, 59, 16594. 10.1002/anie.202005472 32519452

[50] B. Li , Y. Zeng , W. Zhang , B. Lu , Q. Yang , J. Zhou , Z. He , Sci. Bull. 2024, 69, 688. 10.1016/j.scib.2024.01.011 38238207

[51] J. Zhu , M. Yang , Y. Hu , M. Yao , J. Chen , Z. Niu , Adv. Mater. 2024, 36, e2304426. 37555530 10.1002/adma.202304426

[52] Y. Ran , M. Li , H. Zhao , J. Ren , Y. Sheng , G. Shao , Y. Wang , Y. Lei , Adv. Funct. Mater. 2025, 10.1002/adfm.202510241.

[53] K. Wang , R. Yuan , Y. He , S. Reng , Q. Gou , S. Zhang , J. Deng , Z. Luogu , Z. Chen , X. Gu , M. Li , Rare Met. 2024, 44, 912.

[54] J. Zhi , S. Li , M. Han , P. Chen , Sci. Adv. 2020, 6, eabb1342. 32821832 10.1126/sciadv.abb1342 PMC7413738

[55] Y. An , Y. Tian , J. Feng , Y. Qian , Mater. Today 2022, 57, 146.

[56] Y. An , Y. Tian , Q. Man , H. Shen , C. Liu , Y. Qian , S. Xiong , J. Feng , Y. Qian , ACS Nano 2022, 16, 6755. 35357131 10.1021/acsnano.2c01571

[57] Z. Zheng , S. Guo , M. Yan , Y. Luo , F. Cao , Adv. Mater. 2023, 35, e2304667. 37730093 10.1002/adma.202304667

[58] Y. Tan , D. Chen , Y. Liu , Y. Zhang , T. Yao , C. Miao , H. Yang , L. Li , V. Kotsiubynskyi , G. Li , L. Shen , W. Han , J. Energy Chem. 2025, 110, 246.

[59] S. So , Y. N. Ahn , J. Ko , I. T. Kim , J. Hur , Energy Storage Mater. 2022, 52, 40.

[60] S. Zhou , X. Meng , C. Fu , D. Xu , J. Li , Q. He , S. Lin , S. Liang , Z. Chang , A. Pan , Small 2023, 19, 2303457. 10.1002/smll.202303457 37394714

[61] S. Yang , Y. Zhang , Y. Zhang , J. Deng , N. Chen , S. Xie , Y. Ma , Z. Wang , Adv. Funct. Mater. 2023, 33, 2304280.

[62] Y. Liang , D. Ma , N. Zhao , Y. Wang , M. Yang , J. Ruan , G. Yang , H. Mi , C. He , P. Zhang , Adv. Funct. Mater. 2022, 32, 2112936.

[63] Y. Su , B. Liu , Q. Zhang , J. Peng , C. Wei , S. Li , W. Li , Z. Xue , X. Yang , J. Sun , Adv. Funct. Mater. 2022, 32, 2204306.

[64] Y. Wang , Z. Wang , W. K. Pang , W. Lie , J. A. Yuwono , G. Liang , S. Liu , A. M. Angelo , J. Deng , Y. Fan , K. Davey , B. Li , Z. Guo , Nat. Commun. 2023, 14, 2720. 37169771 10.1038/s41467-023-38384-x PMC10175258

[65] L. Cao , D. Li , E. Hu , J. Xu , T. z. Deng , L. Ma , Y. Wang , X. Q. Yang , C. Wang , J. Am. Chem. Soc. 2020, 142, 21404. 33290658 10.1021/jacs.0c09794

[66] D. Li , Y. Tang , S. Liang , B. Lu , G. Chen , J. Zhou , Energy Environ. Sci. 2023, 16, 3381.

[67] L. Jiang , D. Li , X. Xie , D. Ji , L. Li , L. Li , Z. He , B. Lu , S. Liang , J. Zhou , Energy Storage Mater. 2023, 62, 102932.

[68] H. Yang , Y. Qiao , Z. Chang , H. Deng , P. He , H. Zhou , Adv. Mater. 2020, 32, e2004240. 32797719 10.1002/adma.202004240

[69] H. Yang , Z. Chang , Y. Qiao , H. Deng , X. Mu , P. He , H. Zhou , Angew. Chem. Int. Ed. 2020, 59, 9377. 10.1002/anie.202001844 32202034

[70] Y. Zou , Y. Mu , T. Wang , Z. Chen , Z. Shi , W. Guo , T. Shen , Z. Meng , J. Hu , L. Zeng , T. Liu , J. Sun , Angew. Chem. Int. Ed. 2025, 64, e202510080. 10.1002/anie.202510080 40518445

[71] Z. He , X. Zhu , Y. Song , B. Li , X. Xu , Z. Zhang , N. Zhao , Y. Liu , J. Zhu , L. Wang , L. Dai , H. Tian , Energy Storage Mater. 2025, 74, 103886.

[72] H. Ma , J. Yu , M. Chen , X. Han , J. Chen , B. Liu , S. Shi , Adv. Funct. Mater. 2023, 33, 2307384.

[73] R. Li , L. Pan , Z. Peng , N. Zhao , Z. Zhang , J. Zhu , L. Dai , L. Wang , Z. He , J. Energy Chem. 2024, 93, 213.

[74] V. M. Eigen , K. Tamm , März 1962, 2, 107.

[75] Q. Gou , H. Luo , Q. Zhang , J. Deng , R. Zhao , O. Odunmbaku , L. Wang , L. Li , Y. Zheng , J. Li , D. Chao , M. Li , Small 2023, 19, e2207502. 36650991 10.1002/smll.202207502

[76] H. Yang , Y. Qiao , Z. Chang , H. Deng , X. Zhu , R. Zhu , Z. Xiong , P. He , H. Zhou , Adv. Mater. 2021, 33, 2102415. 10.1002/adma.202102415 34338385

[77] J. Zhu , Z. Bie , X. Cai , Z. Jiao , Z. Wang , J. Tao , W. Song , H. J. Fan , Adv. Mater. 2022, 34, e2207209. 36065756 10.1002/adma.202207209

[78] W. Guo , L. Xu , Y. Su , L. Zhao , Y. Ding , Y. Zou , G. Zheng , T. Cheng , J. Sun , Angew. Chem. Int. Ed. 2025, 64, e202417125. 10.1002/anie.202417125 39425461

[79] Q. Meng , Q. Bai , R. Zhao , P. Cao , G. Zhang , J. Wang , F. Su , X. Zhou , J. Yang , J. Tang , Adv. Energy Mater. 2023, 13, 2302828.

[80] Z. Tian , J. Yin , T. Guo , Z. Zhao , Y. Zhu , Y. Wang , J. Yin , Y. Zou , Y. Lei , J. Ming , O. Bakr , O. F. Mohammed , H. N. Alshareef , Angew. Chem., Int. Ed. 2022, 61, e202213757. 10.1002/anie.202213757 36287573

[81] M. Li , X. Wang , J. Meng , C. Zuo , B. Wu , C. Li , W. Sun , L. Mai , Adv. Mater. 2023, 36, 2308628. 10.1002/adma.202308628 37910810

[82] Z. Hu , Z. Song , Z. Huang , S. Tao , B. Song , Z. Cao , X. Hu , J. Wu , F. Li , W. Deng , H. Hou , X. Ji , G. Zou , Angew. Chem., Int. Ed. 2023, 62, e202309601. 10.1002/anie.202309601 37548132

[83] X. Liang , X. Chen , Z. Zhai , T. Yu , H. Yu , H. Wang , D. Meng , L. Peng , S. Yin , Chem. Eng. J. 2024, 493, 152622.

[84] M. Wang , Z. Dai , C. Yang , D. Xu , X. Zhang , L. Que , X. Zhang , J. Qin , Mater. Today Energy 2024, 46, 101736.

[85] J. Cao , D. Zhang , R. Chanajaree , D. Luo , X. Yang , X. Zhang , J. Qin , Chem. Eng. J. 2024, 480, 147980.

[86] W. Yang , W. Yang , Y. Huang , Y. Wu , X. Ma , L. Dong , X. Peng , Energy Storage Mater. 2025, 80, 104436.

[87] H. Qin , W. Chen , W. Kuang , N. Hu , X. Zhang , H. Weng , H. Tang , D. Huang , J. Xu , H. He , Small 2023, 19, e2300130. 36794300 10.1002/smll.202300130

[88] T. Li , Y. Gong , H. Yang , Y. Liu , X. Dong , Y. Xu , H. Ma , C. Wei , S. Zhang , F. Huang , M. Yang , T. Lin , Energy Storage Mater. 2025, 79, 104311.

[89] Y. Dong , W. Fan , X. Wang , H. Huang , Y. Zhu , J. Chen , W. Tian , Y. Huang , J. Wu , Adv. Funct. Mater. 2025, 10.1002/adfm.202513685.

[90] M. Zhou , S. Guo , J. Li , X. Luo , Z. Liu , T. Zhang , X. Cao , M. Long , B. Lu , A. Pan , G. Fang , J. Zhou , S. Liang , Adv. Mater. 2021, 33, 2100187. 10.1002/adma.202100187 33864653

[91] C. Meng , W. He , L. Jiang , Y. Huang , J. Zhang , H. Liu , J. J. Wang , Adv. Funct. Mater. 2022, 32, 2207732.

[92] H. Wang , Y. Chen , H. Yu , W. Liu , G. Kuang , L. Mei , Z. Wu , W. Wei , X. Ji , B. Qu , L. Chen , Adv. Funct. Mater. 2022, 32, 2205600.

[93] T. Wang , J. Sun , Y. Hua , B. N. V. Krishna , Q. Xi , W. Ai , J. S. Yu , Energy Storage Mater. 2022, 53, 273.

[94] X. Yang , C. Li , Z. Sun , S. Yang , Z. Shi , R. Huang , B. Liu , S. Li , Y. Wu , M. Wang , Y. Su , S. Dou , J. Sun , Adv. Mater. 2021, 33, 2105951. 10.1002/adma.202105951 34617348

[95] J. Zheng , Q. Zhao , T. Tang , J. Yin , C. D. Quilty , G. D. Renderos , X. Liu , Y. Deng , L. Wang , D. C. Bock , C. Jaye , D. Zhang , E. S. Takeuchi , K. J. Takeuchi , A. C. Marschilok , L. A. Archer , Science 2019, 366, 645. 31672899 10.1126/science.aax6873

[96] J. Zhou , M. Xie , F. Wu , Y. Mei , Y. Hao , R. Huang , G. Wei , A. Liu , L. Li , R. Chen , Adv. Mater. 2021, 33, e2101649. 34240487 10.1002/adma.202101649

[97] Z. Zheng , X. Zhong , Q. Zhang , M. Zhang , L. Dai , X. Xiao , J. Xu , M. Jiao , B. Wang , H. Li , Y. Jia , R. Mao , G. Zhou , Nat. Commun. 2024, 15, 753. 38272872 10.1038/s41467-024-44893-0 PMC10810881

[98] C. Chao , Y. Li , Y. Zhao , Rare Met. 2024, 43, 4807.

[99] Y. Luo , Y. Yang , Y. Tao , D. Huang , B. Huang , H. Chen , ACS Appl. Energy Mater. 2021, 4, 14599.

[100] J. Cao , D. Zhang , C. Gu , X. Wang , S. Wang , X. Zhang , J. Qin , Z. S. Wu , Adv. Energy Mater. 2021, 11, 2101299.

[101] X. Huo , G. Gao , B. Li , Z. Zhou , K. Shu , J. Bi , Z. Du , L. Xu , W. Ai , Adv. Energy Mater. 2025, 10.1002/aenm.202502238.

[102] K. Yu , Y. Wen , M. Yan , X. Teng , W. Yang , S. Lian , J. Zhang , F. Zhang , X. Jiang , Y. Luo , L. Mai , Mater. Today Energy 2024, 40, 101488.

[103] S. Zheng , X. Yang , D. Chen , S. Huang , C. Zheng , H. Zhong , W. Zhang , X. Xiang , N. Zhang , Y. Sun , L. Liu , Small 2025, 21, e2411463. 39737779 10.1002/smll.202411463

[104] H. Fu , L. Xiong , W. Han , M. Wang , Y. J. Kim , X. Li , W. Yang , G. Liu , Energy Storage Mater. 2022, 51, 550.

[105] Q. Zhu , G. Sun , S. Qiao , D. Wang , Z. Cui , W. Zhang , J. Liu , Adv. Mater. 2023, 36, 2308577. 10.1002/adma.202308577 38091607

[106] Y. Su , L. Xu , Y. Sun , W. Guo , X. Yang , Y. Zou , M. Ding , Q. Zhang , C. Qiao , S. Dou , T. Cheng , J. Sun , Small 2023, 20, 2308209. 10.1002/smll.202308209 37880867

[107] X. Yuan , D. Zhang , H. Lu , Y. Song , Z. Du , M. Song , N. Lyu , Y. Jin , Adv. Sci. 2025, 10.1002/advs.202506035. PMC12462938 40734633

[108] Y. Zhang , G. Yang , M. L. Lehmann , C. Wu , L. Zhao , T. Saito , Y. Liang , J. Nanda , Y. Yao , Nano Lett. 2021, 21, 10446. 34870997 10.1021/acs.nanolett.1c03792

[109] F. Wu , X. Wei , H. Gu , J. Guan , Y. Mu , R. Liao , Y. Chen , X. Wu , M. Han , L. Zeng , Rare Met. 2025, 44, 7173.

[110] Y. Li , X. Peng , X. Li , H. Duan , S. Xie , L. Dong , F. Kang , Adv. Mater. 2023, 35, e2300019. 36787635 10.1002/adma.202300019

[111] L. Yang , Y. J. Zhu , H. P. Yu , Z. Y. Wang , L. Cheng , D. D. Li , J. Tao , G. He , H. Li , Adv. Energy Mater. 2024, 14, 2401858.

[112] D. Ma , F. Li , K. Ouyang , Q. Chen , J. Zhao , M. Chen , M. Yang , Y. Wang , J. Chen , H. Mi , C. He , P. Zhang , Nat. Commun. 2025, 16, 4817. 40410170 10.1038/s41467-025-60190-w PMC12102169

[113] T. Yan , B. Wu , S. Liu , C. Xiang , M. Tao , L. Niu , J. Liang , Z. Cui , L. Du , H. Song , Z. Liang , Nano Res. 2025, 18, 94907601.

[114] H. Ma , H. Chen , M. Chen , A. Li , X. Han , D. Ma , P. Zhang , J. Chen , Nat. Commun. 2025, 16, 1014. 39856065 10.1038/s41467-025-56325-8 PMC11760366

[115] X. Zhu , Z. Xu , T. Zhang , J. Zhang , Y. Guo , M. Shan , K. Wang , T. Shi , G. Cui , F. Wang , G. Xu , M. Zhu , Adv. Funct. Mater. 2024, 34, 2407262.

[116] Y. Dai , C. Zhang , J. Li , X. Gao , P. Hu , C. Ye , H. He , J. Zhu , W. Zhang , R. Chen , W. Zong , F. Guo , I. P. Parkin , D. J. L. Brett , P. R. Shearing , L. Mai , G. He , Adv. Mater. 2024, 36, e2310645. 38226766 10.1002/adma.202310645 PMC11475447

[117] J. D. Feng , W. K. Han , J. J. He , Y. Liu , R. M. Zhu , J. Zhang , H. Pang , Z. G. Gu , Angew. Chem. Int. Ed. 2025, 64, e202506006. 10.1002/anie.202506006 40437656

[118] S. Meng , S. Yang , T. He , J. Guo , M. Xu , L. Chen , K. Liao , L. Zu , C. Zhou , C. Zhang , J. Yang , ACS Nano 2025, 19, 19670. 40399241 10.1021/acsnano.5c00203

[119] Y. Kong , L. Wang , M. Mamoor , B. Wang , G. Qu , Z. Jing , Y. Pang , F. Wang , X. Yang , D. Wang , L. Xu , Adv. Mater. 2024, 36, e2310143. 38134811 10.1002/adma.202310143

[120] H. Bi , D. Tian , Z. Zhao , Q. Yang , Y. Yuan , R. Zhang , L. Ai , X. Wang , J. Qiu , Adv. Funct. Mater. 2025, 35, 2423115.

[121] R. Xue , Z. Wang , N. Yao , Y. Liu , H. Wang , M. Zhang , A. Shao , X. Tang , J. Liu , J. Tang , Z. Wang , Y. Ma , Adv. Funct. Mater. 2024, 34, 2400959.

[122] Y. Qin , X. Wang , Angew. Chem. Int. Ed. 2024, 63, e202315464. 10.1002/anie.202315464 38032352

[123] J. Ma , X. Shi , Z. Wang , L. Zhou , X. Liu , X. Lu , Z. Jiang , Adv. Mater. 2024, 36, e2406429. 39254352 10.1002/adma.202406429

[124] Z. Li , L. Ye , G. Zhou , W. Xu , K. Zhao , X. Zhang , S. Hong , T. Ma , M.‐C. Li , C. Liu , C. Mei , Chem. Eng. J. 2023, 457, 141160.

[125] Y. Zhao , H. Du , Y. Kang , J. Zhang , B. Lan , Z. Guo , M.‐M. Titirici , Y. Zhao , N. Tavajohi , F. Kang , B. Li , Nat. Rev. Mater. 2025, 10.1038/s41578-025-00816-z.

[126] B. K. Biswal , B. Zhang , P. Thi Minh Tran , J. Zhang , R. Balasubramanian , Chem. Soc. Rev. 2024, 53, 5552. 38644694 10.1039/d3cs00898c

[127] L. Yao , G. Wang , F. Zhang , X. Chi , Y. Liu , Energy Environ. Sci. 2023, 16, 4432.

[128] W. Zhang , X. Zhu , L. Kang , Z. Peng , J. Zhu , L. Pan , L. Dai , S. Liu , L. Wang , Y. Liu , Z. He , J. Energy Chem. 2024, 90, 23.

[129] S. Lv , M. Su , Z. Li , Y. Mao , J. Yin , D. Cao , G. Wang , J. Yi , F. Ning , K. Zhu , Adv. Funct. Mater. 2024, 34, 2315910.

